# Statistical learning of target selection and distractor suppression shape attentional priority according to different timeframes

**DOI:** 10.1038/s41598-021-93335-0

**Published:** 2021-07-02

**Authors:** Valeria Di Caro, Chiara Della Libera

**Affiliations:** 1 Department of Neurosciences, Biomedicine and Movement Sciences, University of Verona, Verona, Italy; 2 Section of Physiology and Psychology, Department of Neurosciences, Biomedicine and Movement Sciences, University of Verona – Medical School, Strada Le Grazie 8, 37134 Verona, Italy

**Keywords:** Attention, Human behaviour

## Abstract

Recent findings suggest that attentional and oculomotor control is heavily affected by past experience, giving rise to selection and suppression history effects, so that target selection is facilitated if they appear at frequently attended locations, and distractor filtering is facilitated at frequently ignored locations. While selection history effects once instantiated seem to be long-lasting, whether suppression history is similarly durable is still debated. We assessed the permanence of these effects in a unique experimental setting investigating eye-movements, where the locations associated with statistical unbalances were exclusively linked with either target selection or distractor suppression. Experiment 1 and 2 explored the survival of suppression history in the long and in the short term, respectively, revealing that its lingering traces are relatively short lived. Experiment 3 showed that in the very same experimental context, selection history effects were long lasting. These results seem to suggest that different mechanisms support the learning-induced plasticity triggered by selection and suppression history. Specifically, while selection history may depend on lasting changes within stored representations of the visual space, suppression history effects hinge instead on a functional plasticity which is transient in nature, and involves spatial representations which are constantly updated and adaptively sustain ongoing oculomotor control.

## Introduction

Human behavior can adapt quickly and precisely to meet the ever-changing requests of the surrounding environment, especially with respect to events that occur repeatedly and/or can be predicted from past experience^1,2^. These changes reflect an extraordinary ability of the brain to initiate neural plasticity in response to events and their outcomes, a fundamental feature for several neural circuits across the lifespan^3^. Indeed, it is well-known that since early development stages and onwards, virtually all cognitive functions and the underlying neural circuits are highly sensitive with respect to what is learned through experience, maximizing fitness to the environment^4^.

Distinct systems however may exhibit a different degree of adaptability, and the extent to which plastic changes may last varies. Sometimes changes occur via rapid and dynamic temporary adjustments, as in the circuits involved in brightness adaptation^5^, other times the adjustments, triggered by extensive exposure to crucial stimulation, become hardwired and persistent, as in several forms of learning^6,7^.

In recent years many studies tackled the adaptive features of visual attention, or the crucial set of cognitive functions which aid behavioral planning and execution on the basis of internal goals and external conditions. Guided by different signals, visual attention mechanisms preliminarily process the environment, discriminate relevant vs. irrelevant elements, and enhance the representation of selected objects and locations, so that they become the target of subsequent behavioral responses, i.e., an eye movement that aligns the fovea with the object of interest and/or a hand or limb reaching movement towards it^8–10^.

In the past decade or so it has become increasingly clear that past experience has a paramount importance among the factors controlling attentional deployment. At least three sources of attentional control have been identified: (i) Attention can be reflexively oriented towards conspicuous or unexpected stimuli, in bottom-up; (ii) it can be guided towards items that are relevant for the current goals, irrespectively of their apparent features, in top-down; (iii) it can be allocated towards objects that in the past have been attentionally processed more often or with more beneficial consequences^11–15^.

One of the most powerful factors pertaining to the latter category is statistical learning, a form of implicit learning based on the statistical regularities present in the environment^16–20^. Following repeated exposure to given stimuli within a given task, participants exhibit a facilitated attentional processing of stimuli or spatial locations that have been more frequently attended to. This phenomenon, often assimilated to the formation of a habit^21^, occurs irrespectively of the visual features of the stimuli involved, and irrespectively of their top-down relevance, and has been specifically explored considering how it affects the spatial deployment of attention.

Importantly, these effects seem to equally pervade two distinct aspects of attentional processing, that are the selection of relevant information and the inhibition of the non-relevant one. Both are thought to derive from changes in the strength with which stimulus locations are coded within priority maps of the visual field, or topographically organized representations of the visual environment possibly supported by neural circuits in frontoparietal cortices^22,23^. Several studies have described selection history effects, where repeated exposure to target stimuli at a given location—accordingly selected and responded to—led to benefits in subsequent performance to new targets at the same location. Such implicit learning is thought to trigger, in the priority maps involved during task performance, increases in the baseline activity associated with the frequently selected locations. These shifts in baseline priority are thought responsible for the facilitated processing of any task relevant item appearing therein^14–16,24,25^.

On the other hand, studies have also shown suppression history effects, such that if specific locations in the visual field are more often occupied by non-relevant distracting stimuli they become more easily ignored. This phenomenon is thought to depend on decreases in the baseline activity within the portions of priority maps coding for the locations involved, determined by the accumulation of inhibitory traces left by visual attention when dealing with distractor rejection. Any stimulus appearing at frequently ignored locations will therefore have a lower probability of being processed and will trigger less interference with the ongoing selection of task-relevant information^18,19,26–33^.

Adopting a parsimonious approach, one could conceive that both selection and suppression history hinge on the very same priority maps, by either increasing or decreasing the baseline levels at the nodes coding for the relevant coordinates. In support of this possibility, “transfer effects” have been described, so that the devaluation of a location due to suppression history hampers the selection of targets that appear therein^18,28,34,35^ and selection history increases the interference associated with salient distractors appearing at frequently selected locations^18,36^.

Interestingly, the studies conducted so far on these forms of implicit attentional learning have shown many commonalities. For instance, both lead to robust effects in performance and both are exhibited very rapidly from the onset of stimulus probability unbalances, becoming statistically significant over a handful of trials^18,37–39^. Their lifetime however, and especially their survival after all unbalances in stimulus probability are removed, is less clear and in some cases controversial. Studies have shown that the effects of selection history are persistent and continue to affect performance not only immediately after the learning session^18,40^, but also in the long-term, surviving up to a week after that^37^. These results are in line with literature on other forms of statistical learning, drawing a parallel between learning phenomena across different cognitive domains^41–43^.

On the other hand, the evidence relative to the permanence of suppression history effects is scarce and mixed. While in some cases the effects failed to survive after all frequency biases were removed^18^, in others the permanence of the effects seemed to depend upon the perceptual relationships between targets and distractors in the specific experimental task^30^. Sauter and colleagues for instance observed persistent suppression history effects, both immediately after the learning session and 24 h later, only if targets and distractors were defined by the same perceptual feature (i.e., orientation), and thus deeper perceptual/attentional processing was required to perform, in each trial, a correct selection/filtering between the two^30^.

Overall, the detection of biases in stimulus probability seems to trigger attentional adjustments that respond similarly and symmetrically to unbalances relative to both relevant and irrelevant stimuli. Whether these adjustments will be consolidated however may differ depending on the type of attentional mechanisms that were involved during stimulus processing in the learning sessions, leaving open the possibility that the mechanisms involved in attentional selection and suppression may respond differently to the same experimental manipulations. These issues are quite central within attention research because there is still no definitive consensus on whether target selection and distractor inhibition represent simply two outcomes of a single selection mechanism or whether instead they can be considered as independent mechanisms, possibly hinging on different neural substrates^44^. The observation of similarities or asymmetries in the learning induced by the manipulation of target vs. distractor probabilities could thus provide important information for either of these views.

It must be noted that virtually all studies conducted so far in this domain adopted tasks in which the stimulus locations associated with statistical unbalances could host both target and distractor information^18,19,20,45^. So, for instance, a location more frequently occupied by a target was also occupied by a distractor in other trials, and conversely a location more frequently occupied by a distractor could be occupied by a target in other trials. Each stimulus location therefore was ambiguously tied to both target selection and distractor suppression, and what differed between locations was the relative probability of the two.

To assess more precisely the impact of statistical learning on attentional filtering mechanisms, in a previous study we decided to avoid similar ambiguities so that the locations associated with salient distractors that needed to be filtered out could never be occupied by a task relevant item^38^. We found that salient distractors could be more easily filtered out when they appeared at locations where they were more likely to occur, showing that the priority of spatial locations was affected by suppression history alone, completely decoupled from attentional selection.

**Figure 1:**
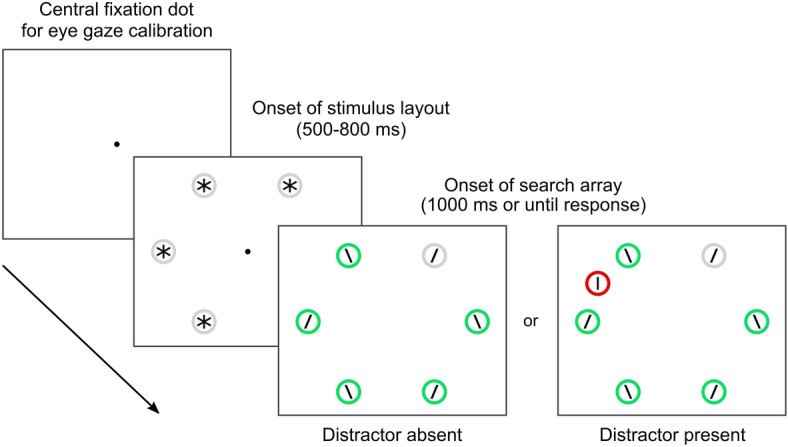
Experimental task and stimuli. Each trial started with a central fixation dot which also served for calibration of eye gaze, then the stimulus layout appeared, which was replaced after a variable time by the search array. The target stimulus in the search array was defined by the circle which remained gray, as opposed to the others which turned green. In a proportion of trials an additional stimulus appeared in a previously empty location, which was irrelevant to the task and acted as a salient distractor.

Following up on our approach, here we explored the extent to which the learning induced by pure suppression history—isolated from selection history—could persist in time, affecting performance after the learning session, when stimulus probabilities cease to be biased. To do so we designed three experiments, in which participants were required to manually discriminate a task relevant stimulus while ignoring a salient distractor. To access the most sensitive correlates of attentional deployment across space^46^ we focused our analyses on the spontaneous eye-movements executed by participants during the task. While eye-movements were never explicitly required they were instrumental to provide a correct response, because the dimension and eccentricity of targets rendered them clearly discriminable only when foveated^38^. They represented therefore a unique window on attentional orienting in a highly controlled and yet ecological context (Fig. 1).

**Figure 2:**
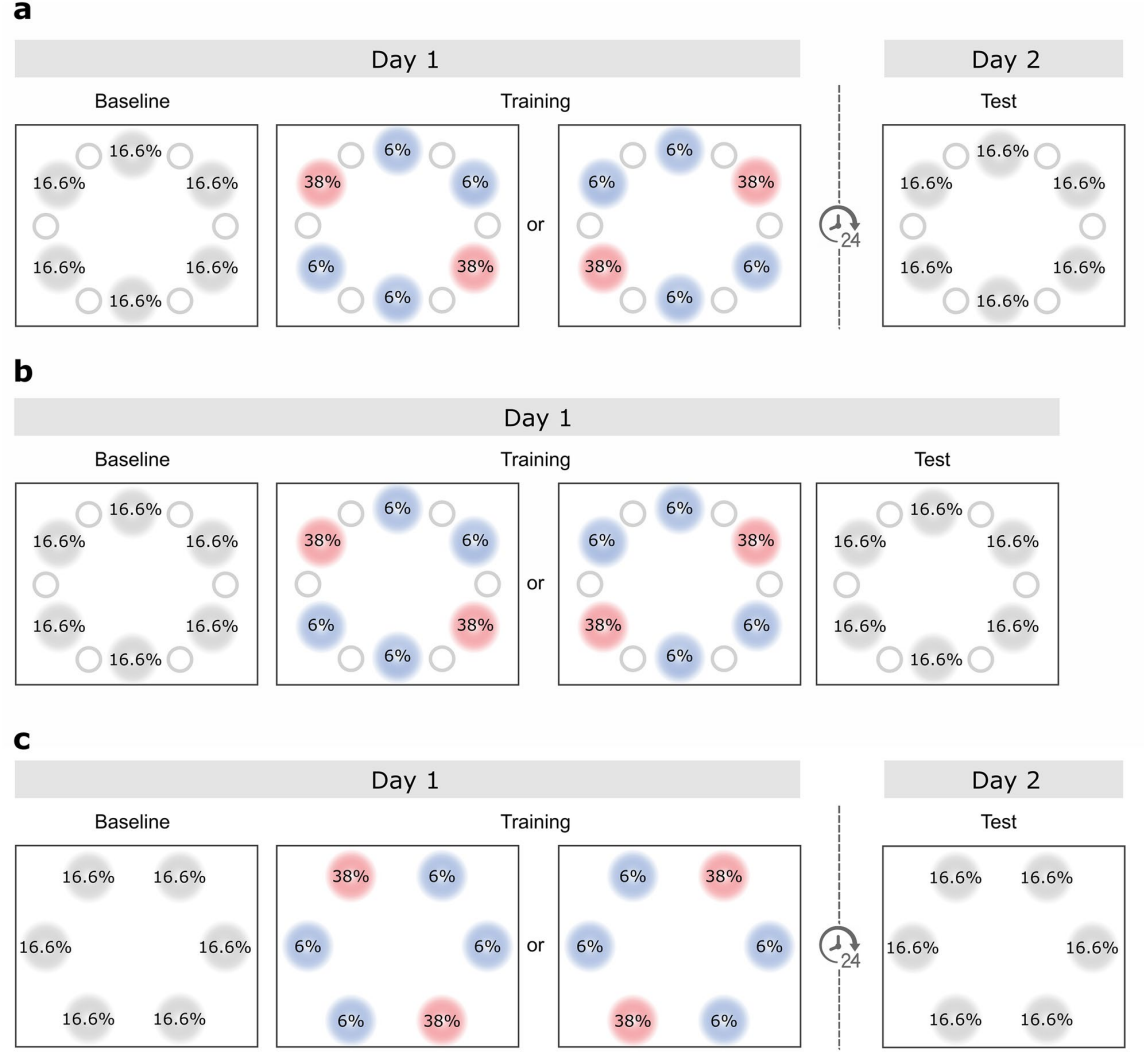
Experimental timeline and stimulus frequency manipulations adopted in the study. Stimulus locations associated with high or low frequency are indicated in red or blue respectively, solely for the purpose of illustration. Locations associated with high stimulus frequency were one on the left and other on the right hemifield, and their specific assignment was counterbalanced across subjects. (**a**) In Experiment 1 Training and Test took place in consecutive days, and the frequency unbalances applied during Training regarded distractor location. (**b**) In Experiment 2 Training and Test took place on the same day within an uninterrupted session, and the frequency unbalances applied during Training regarded distractor location. (**c**) In Experiment 3 Training and Test took place in consecutive days, and the frequency unbalances applied during Training regarded target location.

Each Experiment comprised a Baseline phase, without frequency biases in either target or distractor location, which was immediately followed by a Training, in which frequency unbalances were introduced. In Experiment 1 (Fig. 2a) and 2 (Fig. 2b) we manipulated the probability of distractor location, so that the salient distractor appeared more frequently at two locations in the display. In Experiment 3 instead we manipulated the probability of target location (Fig. 2c), so that the task relevant stimulus was likely to appear in two positions in the display. Finally, the Test phase took place, in which all frequency unbalances were removed. Importantly, while the Test sessions of Experiment 1 and 3 took place 24 h after the end of Training, the Test session of Experiment 2 was carried out immediately after the Training.

While the first two experiments aimed at exploring the survival of suppression history in the long and in the short term, respectively, the third probed the permanence of selection history effects, when—as in our experimental paradigm—it was isolated from a concurrent suppression history at the same locations and allowed to assess the generalizability of our findings relatively to other methodological approaches.

## Results

Statistical analyses considered the first saccadic eye-movement upon search array onset in each trial^32,33,38^. Dependent variables were the percentages of target- and distractor-directed saccades in the conditions of interest (see “Methods” for details on saccades classification based on their endpoint). Given the robust concordance between the two analyses, here we focus on target-directed saccades, while distractor-directed saccades are discussed in Supplementary materials (Supplement 1). For completeness, the overall analyses of manual discrimination responses are also reported in Supplementary materials (Supplement 2). Eye-movements analyses were only performed for trials in which subjects responded correctly. Holm-Bonferroni correction was systematically applied to all multiple t-tests, and the p values reported are adjusted accordingly. When mean values are reported they are accompanied by standard errors in parentheses.

### Experiment 1

#### Baseline

**Figure 3:**
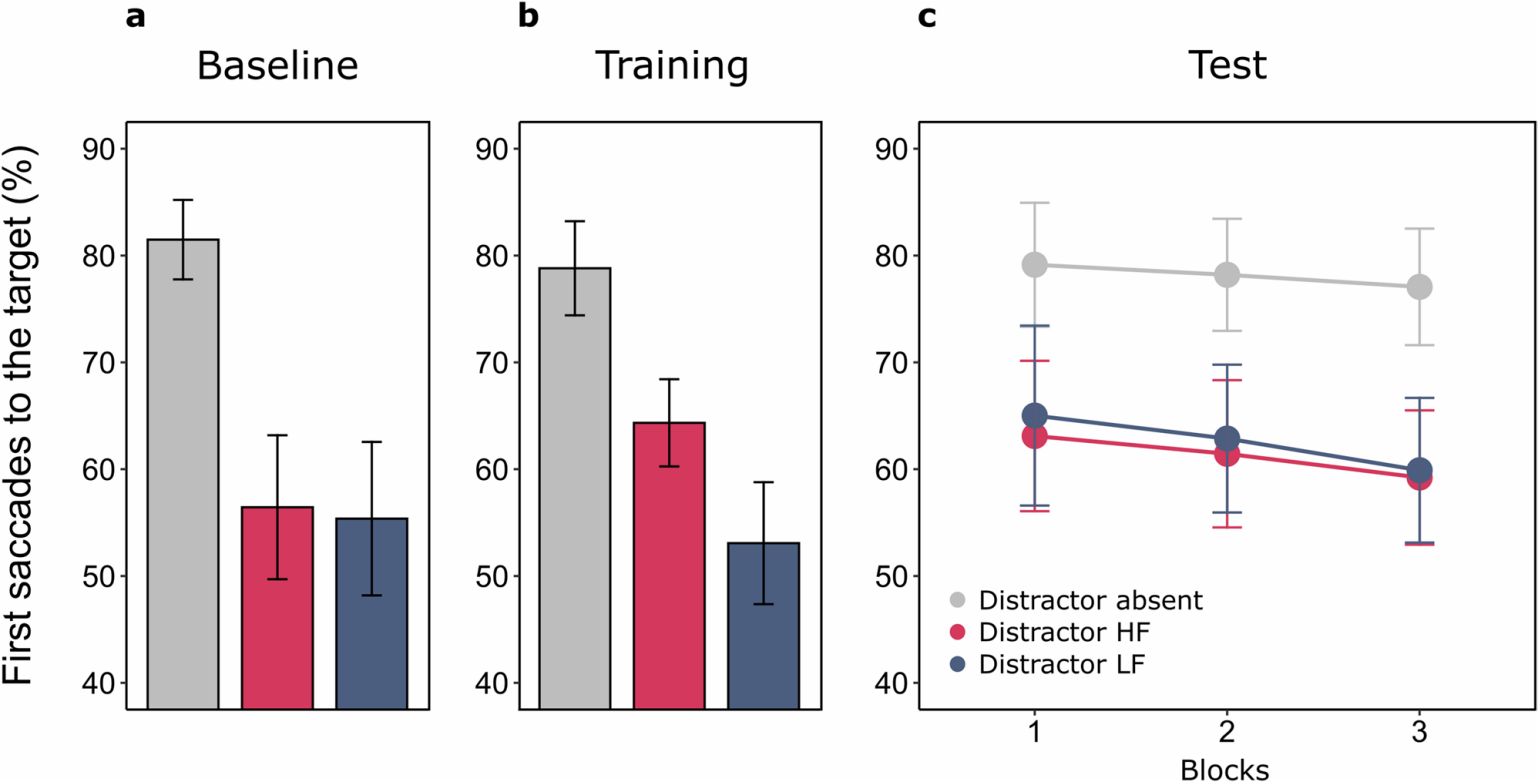
Mean percentages of target-directed saccades as a function of Distractor condition in Experiment 1. (**a**) Performance at baseline. (**b**) Performance at training. (**c**) Performance at test. Here and in all the other Figures, error bars depict the 95% within-subject confidence intervals^47,48^.

Preliminary analyses assessed the overall impact of salient distractors, and performance associated with those at locations that would be manipulated during the Training phase. Distractors reduced significantly the amount of saccades directed to the target, absent Mean 81.5% (SEM ± 2.61), present 55.9% (± 3.67), *t*(17) = 7.629, *p* < 0.001, *d*_*z*_ = 1.798. No differences were found associated with distractor location, HF 56.4% (± 3.72), LF 55.4% (± 4.06), *t*(17) = 0.413, *p* = 0.685, *d*_*z*_ = 0.097 (Fig. 3a).

#### Training phase

The mean percentages of target-directed saccades were submitted to a one-way ANOVA with Distractor location as main factor (absent, present in High Frequency location, or HF, present in Low Frequency location, or LF). The effect of Distractor location was significant, absent 78.8% (± 2.21), HF 64.3% (± 3.07), LF 53.1% (± 3.65), *F*(2,34) = 55.892, *p* < 0.001, ƞ_p_^2^ = 0.767, and post-hoc t-tests confirmed that while a salient distractor reduced the number of first saccades in general (absent vs. HF: *t*(17) = 6.534, *p* < 0.001, *d*_*z*_ = 1.54; absent vs. LF: *t*(17) = 8.218, *p* < 0.001, *d*_*z*_ = 1.937), distractors appearing at high frequency locations were less interfering (HF vs. LF: *t*(17) = 6.341, *p* < 0.001, *d*_*z*_ = 1.495) (Fig. 3b).

#### Test phase

To explore in detail the crucial effects in time, the Test phase was divided in three consecutive blocks, and an ANOVA was conducted with Distractor location (absent, HF, LF) and Block (1 to 3) as within-subjects factors. The effect of Distractor location was significant, absent 78.1% (± 4.84), HF 61.3% (± 4.77), LF 62.6% (± 4.71), *F*(2,34) = 33.582, *p* < 0.001, ƞ_p_^2^ = 0.664, and post-hoc tests suggested, surprisingly, that while distractors always impaired performance (absent vs. HF: *t*(17) = 6.608, *p* < 0.001, *d*_*z*_ = 1.557; absent vs. LF: *t*(17) = 7.074, *p* < 0.001, *d*_*z*_ = 1.667), the difference between the two crucial conditions was no longer significant (HF vs. LF: *t*(17) = 0.639, *p* = 0.531, *d*_*z*_ = 0.151) (Fig. 3c). Neither Block, *F*(2,34) = 2.571, *p* = 0.091, ƞ_p_^2^ = 0.131, nor its interaction with Distractor location, *F*(4,68) = 0.200, *p* = 0.937, ƞ_p_^2^ = 0.012, were significant. So, while the suppression history associated with specific distractor locations remarkably affected eye-movements during Training, when all frequency unbalances were in place, its traces were no longer detectable 24 h later, when all unbalances were removed.

#### Training vs. test

To compare directly Training and Test phases we conducted an ANOVA with Phase (Training vs. Test) and Distractor location (absent, HF, LF) as factors. Performance in each phase was therefore averaged according to each Distractor location condition. Results showed a non-significant effect of Phase, *F*(1,17) = 0.215, *p* = 0.649, ƞ_p_^2^ = 0.012, a significant effect of Distractor location (in line with the main ANOVAs), absent 78.5% (± 3.14), HF 62.9% (± 3.36), LF 57.9% (± 3.32), *F*(2,34) = 58.419, *p* < 0.001, ƞ_p_^2^ = 0.775, and a significant interaction, *F*(2,34) = 13.472, *p* < 0.001, ƞ_p_^2^ = 0.442, stressing once again the qualitative and quantitative difference between performance in the two phases (HF vs. LF across phases, location effect at Training 5.63% (± 0.88), location effect at Test − 0.647% (± 1.07): *t*(17) = 4.204, *p* < 0.001, *d*_*z*_ = 0.991).

To examine in detail the possibility that at least some effects of suppression history might be visible at the beginning of the Test we compared performance at Training with Test block 1, separately for trials with distractors in HF and LF locations. Target-directed saccades in HF trials were indistinguishable between Training and Test, Training HF 64.3% (± 3.06), Test HF63.1% (± 4.76), *t*(17) = 0.257, *p* = 0.800, *d*_*z*_ = 0.061, while the impact of distractors at LF locations was lower at Test, although the difference did not reach statistical significance, Training 53.0% (± 3.63), Test 65.0% (± 5.10): *t*(17) = 2.141, *p* = 0.094, *d*_*z*_ = 0.504.

#### Discussion

This Experiment explored whether the effects of suppression history, established during a learning session with biased distractor probability for specific spatial locations, would survive in the long term, and affect eye-movements after a 24-h delay in a context in which probability unbalances were no longer active. The results of Training phase replicate our previous findings^38^ and are in line with the relevant literature^18–20^, thus proving the establishment of robust suppression history effects. However, no effects were maintained on the following day in the very same experimental task.

Different considerations can be made at this point. First of all, although our distractors had a very high intrinsic saliency and triggered a strong attentional and oculomotor capture (see Supplement 1)^49,50^, performance in similar tasks is known to improve with practice^51,52^. Indeed, leaving aside distractor frequency manipulations, the impact of distractors was reduced on the second day (distractor absent vs present, Training vs. Test: *t*(17) = 2.207, *p* = 0.041, *d*_*z*_ = 0.520). The target-directed saccades observed during Test in distractor-present conditions could then represent a ceiling performance, beyond which there could be no further improvements. If such optimization affected equally performance with both HF and LF distractors, it would hinder any distinction between the two conditions at Test, even if the differential traces of suppression history had been consolidated. Although this is theoretically possible, our results are yet in line with previous evidence suggesting that traces of suppression history might not survive in the long term^18,30^.

Overall, this experiment confirmed that attentional and oculomotor control take advantage of suppression history to reduce the impact of salient distractors. Suppression history associated with specific locations however seems to determine transient benefits that disappear after a 24-h delay.

In Experiment 2 we investigated whether any effects could be observed if the Test phase took place on the same day as Training, by abolishing the delay between the two sessions.

### Experiment 2

#### Baseline

**Figure 4:**
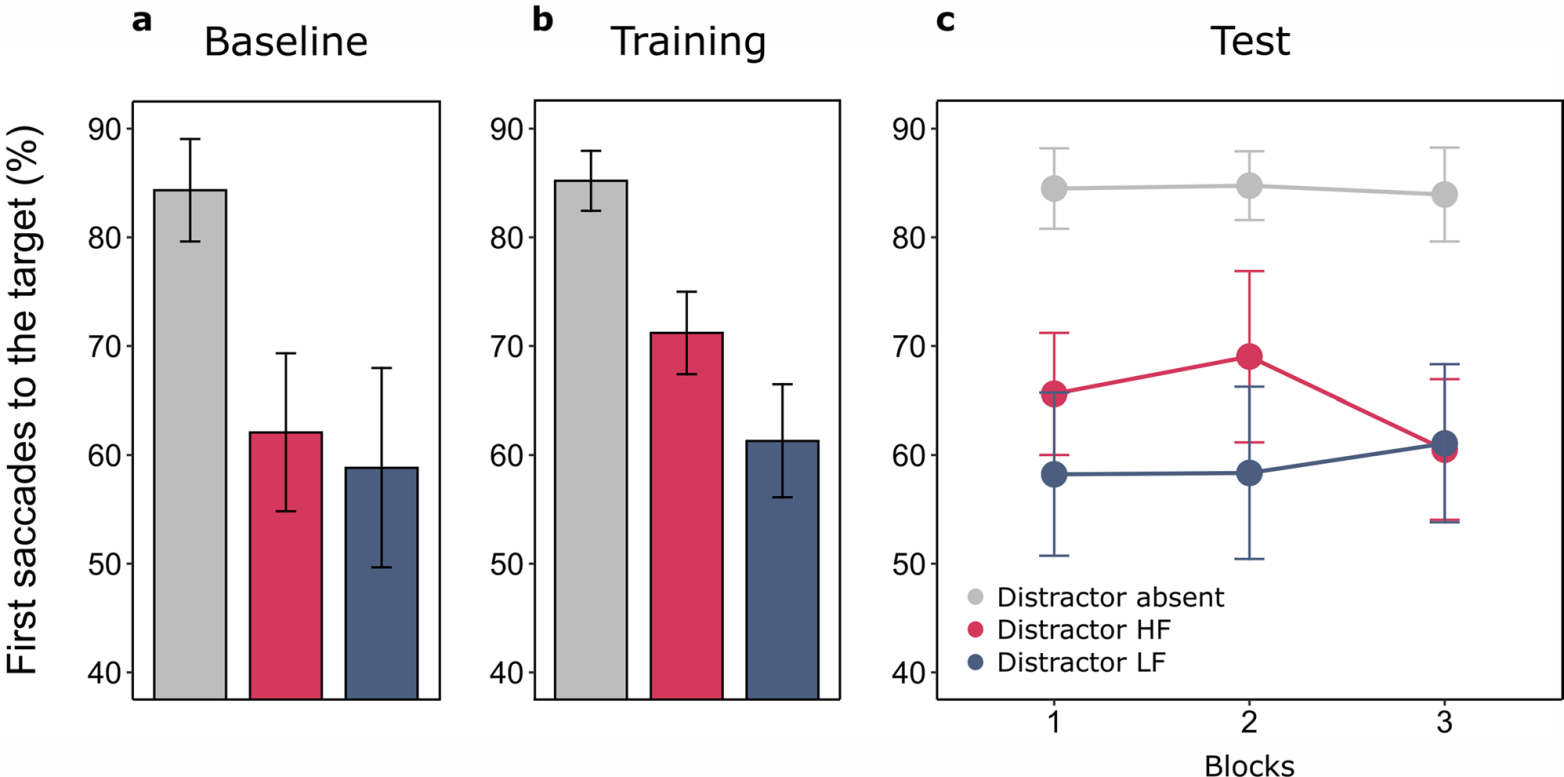
Mean percentages of target-directed saccades as a function of Distractor condition in Experiment 2. (**a**) Performance at baseline. (**b**) Performance at training. (**c**) Performance at test.

The impact of distractors was robust and significant, absent 84.3% (± 2.93), present 60.5% (± 4.37), *t*(19) = 7.203, *p* < 0.001, *d*_*z*_ = 1.611. No significant differences were found in trials with distractors at locations later associated with high or low frequency, HF 62.1% (± 4.63) vs. LF 66.7% (± 5.68), *t*(19) = 0.585, *p* = 0.565, *d*_*z*_ = 0.131) (Fig. 4a).

#### Training phase

The one-way ANOVA on target-directed saccades revealed a significant main effect of Distractor location, absent 85.2% (± 1.82), HF 71.2% (± 2.56), LF 61.3% (± 3.03), *F*(2,38) = 72.976, *p* < 0.001, ƞ_p_^2^ = 0.793, and post-hoc t-tests replicated exactly the results of the Training phase in Experiment 1, once again demonstrating that oculomotor performance, while being impaired by the presence of a distractor (absent vs. HF: *t*(19) = 7.609, *p* < 0.001, *d*_*z*_ = 1.702; absent vs. LF: *t*(19) = 9.203, *p* < 0.001, *d*_*z*_ = 2.058), was significantly better when this appeared at HF locations (HF vs. LF: *t*(19) = 7.534, *p* < 0.001, *d*_*z*_ = 1.685) (Fig. 4b).

#### Test phase

Strikingly, the ANOVA on saccades during the Test phase highlighted a significant main effect of Distractor location, absent 84.4% (± 1.88), HF 65.0% (± 2.67), LF 59.2% (± 3.31), *F*(2,38) = 61.838, *p* < 0.001, ƞ_p_^2^ = 0.765. The main effect of Block, *F*(2,38) = 0.475, *p* = 0.626, ƞ_p_^2^ = 0.024, and the interaction between Block and Distractor location were instead non-significant, *F*(4,76) = 0.930, *p* = 0.451, ƞ_p_^2^ = 0.047 (Fig. 4c). So, in line with Training, distractors continued to have a significant impact on saccadic endpoints (absent vs. HF: *t*(19) = 8.558, *p* < 0.001, *d*_*z*_ = 1.914; absent vs. LF: *t*(19) = 9.590, *p* < 0.001, *d*_*z*_ = 2.144), but their effect was less detrimental if they appeared at HF locations (HF vs. LF: *t*(19) = 2.647, *p* = 0.016, *d*_*z*_ = 0.592).

These results, supported by converging evidence in distractor-directed saccades (Supplement 1), show that although distractor frequency had become equal across the available locations in the display, oculomotor behavior was still exhibiting the suppression history effects triggered during the Training phase. Distractors at locations with a significant history of suppression continued to be ignored more efficiently, on one hand giving rise to lower oculomotor capture (Supplement 1), and on the other by facilitating saccades directed to the target in the array, despite the presence of a salient irrelevant event.

#### Training vs. test

The ANOVA with Phase and Distractor location as factors resulted in a significant effect of Distractor location, absent 84.8% (± 1.68), HF 68.3% (± 2.36), LF 60.1% (± 2.88), *F*(2,38) = 96.228, *p* < 0.001, ƞ_p_^2^ = 0.835, and non-significant effects of Phase, *F*(1,19) = 3.177, *p* = 0.091, ƞ_p_^2^ = 0.143, and Distractor location by Phase, *F*(2,38) = 2.016, *p* = 0.147, ƞ_p_^2^ = 0.096. Indeed, the effects of suppression history matured during Training continued to affect target-directed saccades, optimizing performance in trials with distractors in HF locations, as the impact of distractor location did not differ significantly (HF vs. LF effect across Phases, *t*(19) = 1.621, *p* = 0.121, *d*_*z*_ = 0.363). The same conclusion could be derived from the analyses on distractor-directed saccades (Supplement 1).

#### Discussion

Experiment 2 explored whether the effects of suppression history were found when frequency unbalances were removed immediately after the learning session. In a new group of participants, we replicated the basic suppression history results^38^, once again confirming that eye-movements are readily adjusted when distractor frequency is biased across the visual space, as in the Training phase. Moreover, the consequences of such unbalances continued to affect oculomotor control even when distractor frequency was reset to being equal at all locations. Interestingly, the visual inspection of the data obtained during the Test phase (Fig. 4c) seemed to suggest that, perhaps with a longer session, the impact of suppression history might have eventually faded, as a likely result of continuous readjustments of space-based priority triggered by updating stimulus probabilities. These findings, together with those of Experiment 1, suggest that the history of distractor suppression, manipulated in isolation from other forms of attentional processing at the same locations, leads to adaptations that are remarkably dynamic, respond quickly and efficiently to any unbalances in stimulus probability and affect only immediate behavior, leaving traces that do not appear to be consolidated in long-term memory. These results seem in partial contrast with previous literature on statistical learning of distractor suppression, which however stems from crucially different experimental paradigms. As already explained, in our displays the locations occupied by targets and distractors were never coinciding. Distractor-related locations therefore were never task relevant, and the only attentional operation involving stimuli presented therein was distractor suppression. Before discussing further the implications of our findings we decided to run a new experiment to evaluate whether our paradigm could, in principle, lead to lasting effects of statistical learning when target frequency was manipulated.

### Experiment 3

#### Baseline

**Figure 5:**
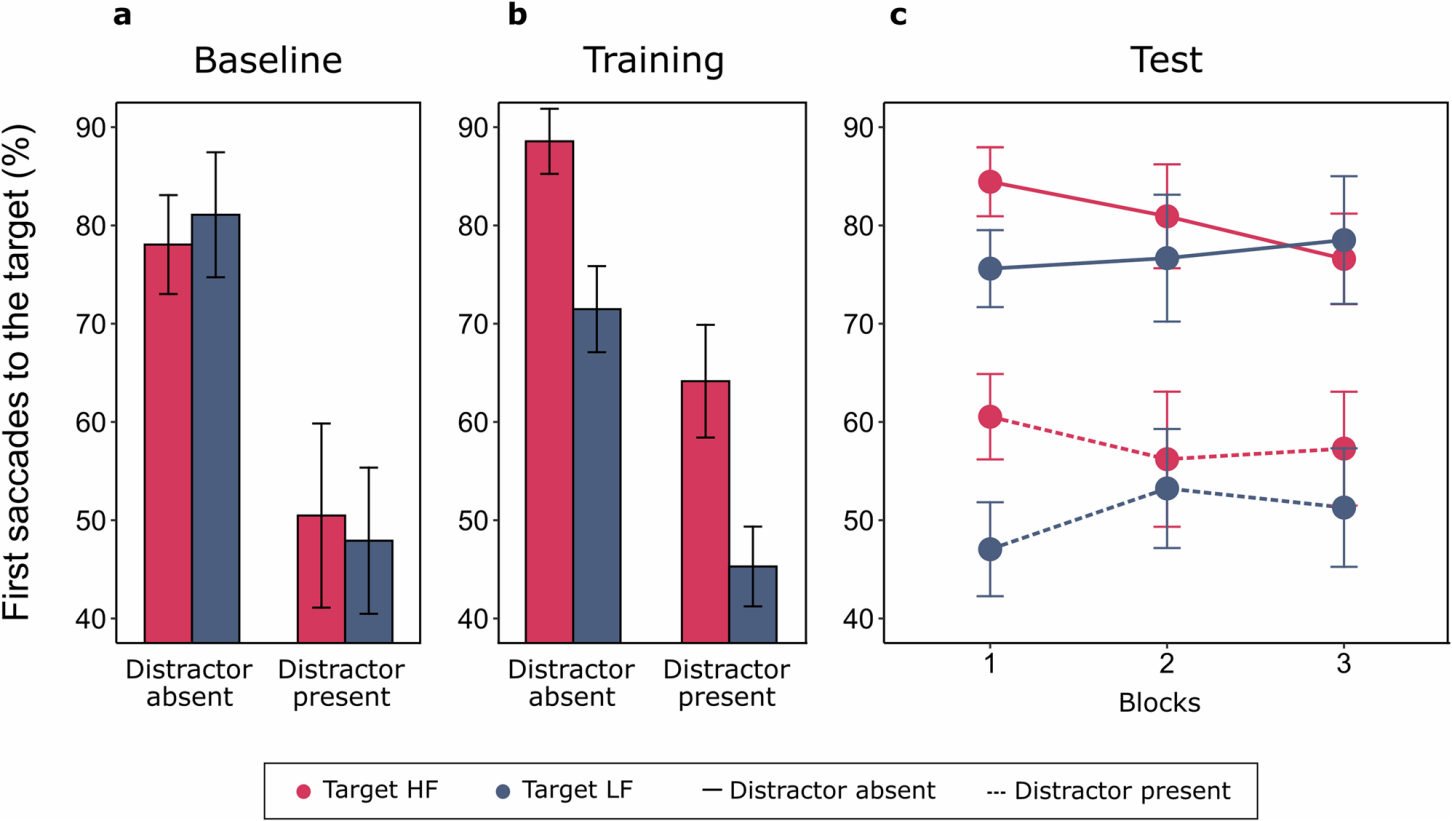
Mean percentages of target-directed saccades as a function of Target and Distractor conditions in Experiment 3. (**a**) Performance at baseline. (**b**) Performance at training. (**c**) Performance at test.

Once again, the impact of distractors on target-directed saccades was significant, absent 79.6% (± 3.04), present 49.2% (± 5.33), *t*(18) = 9.107, *p* < 0.001, *d*_*z*_ = 2.089, and no significant effects were found considering trials with targets appearing at locations later associated with frequency manipulations, HF 64.3% (± 4.80) vs. LF 64.5% (± 4.40), *t*(18) = 0.052, *p* = 0.959, *d*_*z*_ = 0.012 (Fig. 5a).

#### Training phase

The ANOVA conducted considered Target location (HF vs. LF) and Distractor presence (present vs. absent) as within-subjects factors. The significant effect of Target location, HF 76.4% (± 2.77), LF 58.4% (± 3.91), *F*(1,18) = 52.922, *p* < 0.001, ƞ_p_^2^ = 0.746, indicated that targets at HF locations received a significantly higher amount of first saccades. The main effect of Distractor presence was also significant, absent 80.0% (± 2.67), present 54.7% (± 4.19), *F*(1,18) = 66.991, *p* < 0.001, ƞ_p_^2^ = 0.788, underlining once more the detrimental impact of distractors. Interestingly however, no significant interaction was found between Target location and Distractor presence, *F*(1,18) = 0.314, *p* = 0.582, ƞ_p_^2^ = 0.017, indicating that while the ongoing unbalances in target frequency were effective in optimizing oculomotor behavior and prioritize spatial locations more frequently occupied by task-relevant stimuli, this effect was dissociated from the attentional filtering of distractors, that when present led to a systematic cost, unaffected by the concurrent frequency manipulations (Fig. 5b). Interestingly, a significant interaction between Target location and Distractor presence emerged instead in the analysis of manual RTs (Supplementary 2), suggesting that Target location effects were larger in Distractor present trials, that overall received slower responses.

#### Test phase

The ANOVA conducted on eye-movements at Test considered Target location (HF vs. LF), Distractor presence (absent vs. present) and Block (1 to 3) as within-subjects factors. Crucially, the main effect of Target location was significant, highlighting that selection history effects were maintained for at least one day after the learning session, HF 69.3% (± 3.51), LF 63.7% (± 3.06), *F*(1,18) = 6.570, *p* = 0.020, ƞ_p_^2^ = 0.267. A significant interaction with Block, *F*(2,36) = 3.269, *p* = 0.050, ƞ_p_^2^ = 0.154, further suggested that this effect of Target location tended to decrease systematically as the session proceeded (HF vs. LF in Block 1: HF 72.5% (± 3.67) vs LF 61.3% (± 3.26), *t*(18) = 4.037, *p* = 0.002, *d*_*z*_ = 0.926; Block 2: HF 68.6% (± 3.86) vs LF 65.0% (± 3.18), *t*(18) = 1.056, *p* = 0.609, *d*_*z*_ = 0.242; Block 3: HF 66.9% (± 3.85) vs LF 64.9% (± 3.87), *t*(18) = 0.661, *p* = 0.609, *d*_*z*_ = 0.151; Target location effect, Block 1 vs. 2: *t*(18) = 2.002, *p* = 0.121, *d*_*z*_ = 0.459; 1 vs. 3: *t*(18) = 2.534, *p* = 0.062, *d*_*z*_ = 0.581; 2 vs. 3: *t*(18) = 0.388, *p* = 0.703, *d* = 0.089). The main effect of Distractor presence was significant, absent 78.8% (± 2.43), present 54.3% (± 4.16), *F*(1,18) = 77.410, *p* < 0.001, ƞ_p_^2^ = 0.811, but its interactions with Target location, *F*(1,18) = 1.809, *p* = 0.195, ƞ_p_^2^ = 0.091, or Block, *F*(2,36) = 0.516, *p* = 0.601, ƞ_p_^2^ = 0.028, were not. Finally, the main effect of Block, *F*(2,36) = 0.178, *p* = 0.838, ƞ_p_^2^ = 0.010, and the interaction comprising all factors were non-significant, *F*(2,36) = 0.840, *p* = 0.440, ƞ_p_^2^ = 0.045 (Fig. 5c).

#### Training vs. test

The ANOVA comprised Phase, Target location and Distractor presence as within-subjects effects. The main effects of Target location, HF 73.1% (± 2.99), LF 60.9% (± 3.38), *F*(1,18) = 44.696, *p* < 0.001, ƞ_p_^2^ = 0.713, and Distractor presence, absent 79.5% (± 2.48), present 54.5% (± 4.08), *F*(1,18) = 76.939, *p* < 0.001, ƞ_p_^2^ = 0.810, were both significant. Interestingly, the interaction between Target location and Phase was also significant, *F*(1,18) = 16.126, *p* < 0.001, ƞ_p_^2^ = 0.473, suggesting that the overall impact of selection history was significantly lower on the second day of practice (HF vs. LF across phases: Target location effect at Training 17.97% (± 2.47), at Test 6.30% (± 2.17), *t*(18) = 4.016, *p* < 0.001, *d*_*z*_ = 0.921). None of the other main effects or interactions were significant (Phase: *F*(1,18) = 0.264, *p* = 0.613, ƞ_p_^2^ = 0.014; Distractor presence x Target location: *F*(1,18) = 1.732, *p* = 0.205, ƞ_p_^2^ = 0.088; Distractor presence x Phase: *F*(1,18) = 0.242, *p* = 0.629, ƞ_p_^2^ = 0.013; Distractor presence x Target location x Phase: *F*(1,18) = 0.310, *p* = 0.584, ƞ_p_^2^ = 0.017).

#### Discussion

The results of Experiment 3 suggest that our paradigm, where target selection and distractor suppression occur at locations that are systematically uncoupled can indeed give rise to selection history effects. These, in line with previous studies on selection history^37^, persist in the long term, affecting performance after a 24-h delay. However, as the session with no frequency unbalances proceeded, the effects tended to disappear, suggesting that this type of attentional learning is highly sensitive to updating evidence on frequency unbalances, and rapidly adapts to new stimulus probabilities.

## General discussion

In a series of experiments, we investigated the durability of statistical learning effects in the attentional domain, specifically those concerning the suppression of salient non relevant visual stimuli. In a visual search task subjects had to discriminate a target which—in a substantial portion of trials—was accompanied by the onset of a very salient event, which was irrelevant and therefore acted as a distractor^49,50,53,54^. The features of this distractor rendered it responsible of generalized costs in performance because it gave rise to an efficient attentional and oculomotor capture (Supplement 1), so that its presence slowed down responses (Supplement 2) and reduced the number of first saccades towards the target.

Task performance under these circumstances depends on the participants’ ability to ignore the distractor, which in turn is allowed by the suppression of its perceptual/attentional processing put in place through inhibitory mechanisms within selective attentional functions. When attentional suppression is frequently associated with some spatial locations, performance can systematically benefit from these contingencies, becoming less sensitive to distractors appearing therein. Although the neural underpinnings of these adaptations have yet to be fully understood^55–60^, they are often conceived as learning-induced adjustments due to the accumulation of inhibitory traces at relative coordinates within spatial representations of the visual space that aid attentional guidance. Much as it happens in the development and consolidation of habits^21,61^, theoretically, the changes induced via such experience-dependent plasticity could be consolidated and, in the absence of new learning, affect performance indefinitely thereafter.

As explained in the Introduction, the evidence is mixed about this possibility, and while some studies suggest that the traces left by distractor inhibition are durable, others cannot support this conclusion^18,30,35^. In our experiments we manipulated the frequency of distractor suppression at given locations in the visual field in such a way that it was completely independent, and systematically decoupled, from other attentional processes, i.e., those involved in target selection. The spatial locations associated with differential suppression history were never occupied by task relevant stimuli, therefore the only traces that could be associated with their representation were those generated by distractor inhibition.

Despite this peculiarity, our experimental paradigm gave rise to robust statistical learning effects^38^, which are comparable to those observed in other experimental contexts. Both selection history (Experiment 3) and suppression history (Experiments 1 and 2) emerged systematically with the introduction of unbalances in stimulus frequency associated with given locations in the visual field.

However, while the effects of selection history seemed to be relatively long lasting, resembling the findings emerged in other more traditional experimental settings^18,37,40^, the effects of pure suppression history were remarkably short-lived. In particular, no lingering effects were found 24 h after the end of the learning session with stimulus unbalances. The statistical contingencies associated with distractor suppression affected the priority of spatial locations only immediately after the learning session, when distractor probability returned to be equal across locations within a continuous flow of trials, so that subjects did not experience a break in between. Even in this case, the effects observed during the test session exhibited a trend which suggested that they would have eventually vanished as the unbiased session proceeded. As a matter of fact, these residual, lingering effects of suppression history could only be appreciated in oculomotor behavior (as reported above and in Supplement 1), while in the RTs of manual task responses they were already non-significant at this time (Supplement 2), a finding which hints to a higher sensitivity of eye-movement measures in detecting residual effects of statistical learning in similar tasks.

Recent research has proposed that the permanence of suppression history effects might depend on the processing depth required by the target/distractor discrimination during task performance^28,30^. These findings have been conceptualized within the Dimension-Weighting Account of visual attention^62–64^, according to which the visual field is represented by means of separate maps, specific for each low-level stimulus dimension (e.g., color, orientation, luminance, size), which convey signals to a superordinate master map. This map encodes saliency by weighting the signals coming from each dimension on the basis of its task-relevance, and eventually guides attentional deployment. According to this view, the deeper is the processing instantiated upon every trial to select relevant from non-relevant information, the stronger will be the traces left by the suppression episode, and the higher will be the probability that these traces will be consolidated in the long term. In these studies, targets and distractors in each display were small bars defined by the same perceptual feature, i.e., orientation. While targets were defined by task instructions, the fact that distractors also exhibited a salient (though irrelevant) orientation, among an otherwise homogeneous background made them stand out and interfere with task performance. Differently from when interference was caused by distractor saliency per se (e.g., when the distractor stood out because it was in a different color), the need to discriminate between task-relevant and irrelevant information at the level of the same feature obliged both pieces of information to be initially selected by attentional mechanisms, to allow a more advanced and detailed analysis at which point the distractor could be eventually discarded and its further processing inhibited. Interestingly, while the effects of suppression history were visible for all kinds of distractors while distractor frequencies were manipulated, they were only found in the long term if this higher degree of perceptual/attentional processing had to be involved during the learning phase. The traces left in place during distractor filtering under these conditions are much more complex than those relative to their inhibition, and comprise a wealth of other types of processing, including a preliminary attentional selection based on their perceptual features^31^. Interestingly, recent studies have shown that in similar visual search tasks, if the perceptual features defining distractors are consistently used across trials (i.e., distractors appear more frequently in the same color or at the same location), the facilitation associated with their filtering is crucially linked with adaptations in the cortical activity of brain areas involved in visual processing as early as V1^59,60^. The reduced interference determined by these distractors might thus be justified by their impoverished low-level perceptual processing, which may be triggered by the detection of intertrial regularities in stimulus properties^60^.

Other studies however have shown that when targets and distractors are systematically associated with highly distinctive perceptual features and locations, the neural correlates of statistical learning for visual stimuli, i.e., a reduction in the activity associated with a stimulus which is highly expected to appear at a given location in the visual field, are only observed if the stimulus in question is task-relevant, and must be attended to. No such adjustments are found if the stimulus, expected on the basis of past experience, is clearly not relevant for the task at hand^65^. Thus, at least for stimuli that can be clearly and unambiguously classified as task relevant or irrelevant, not only they are treated differently from an attentional point of view, being selected or filtered out accordingly, but they also trigger different types of learning-based adjustments, which may be supported by independent neural correlates.

Distractors in our task are unique on many levels: because of their spatial location, their color and their temporal dynamics, appearing abruptly in a previously empty space. Distractor filtering in this context could thus occur at very early stages of stimulus processing, shielding all subsequent target processing steps from being flooded by irrelevant interfering information. While there could be differences due to the specific experimental paradigm in use, when probability unbalances are detected, for instance suggesting that distraction is more frequent at certain spatial coordinates, ongoing performance could be optimized perhaps by engaging proactive inhibitory suppression which prevents any in-depth processing where distraction is expected^66^. Along these lines, studies have shown that when distractor location is cued in advance, and it can be therefore expected explicitly, such shielding can occur proactively, and is associated with specific neural correlates^67–71^.

The emphasis in this field of research is typically put on the flexibility with which attentional processes can be adjusted, whether in top-down^66^ or based on low-level perceptual adaptations^60^, maximizing target selection and distractor filtering according to instructions and explicit knowledge on stimulus probabilities. No speculations are made with respect to whether the effects of such adaptations may be long lasting, although given that the underlying concept is that they are put in place to support ongoing behavior, according to instructions that are by definition only valid within the given experimental session and trials, there seems to be no need for these to be stored in long term and be allowed to affect behavior in the future.

Suppression history effects are largely implicit, as they are triggered by probability biases of which participants are unaware^14,18,19,38^. Perhaps this phenomenon has led to consider these effects akin to other forms of implicit learning due to statistical contingencies, which, as in perceptual and procedural learning, typically imply lasting plasticity in the underlying neural circuits, and support durable changes in future performance^6,7,72^. Our data are in line with a different possibility that the statistical learning of distractor suppression per se may depend on extremely flexible top-down processes, which are triggered and continuously adjusted by unbalances in stimulus probabilities, that shape moment-to-moment attentional deployment across space with the precise aim of maximizing current behavior.

Indeed, there is evidence that many areas in the brain are crucially involved in a predictive type of processing which, based on contextual cues gathered across time and/or space, allow a probabilistic estimation of information that will be relevant both with respect to the perception of sensory input^73^ as well as for the planning and execution of movement^74^. These regions encompass a wide neural network comprising cortical areas in the middle and inferior frontal gyri, premotor cortices, pre-supplementary motor areas, anterior insulae, and temporo-parietal junctions; subcortical structures such as striatum and thalamus as well as the cerebellum^75^.

Interestingly, studies investigating the neural correlates of statistical learning have shown that these comprise both regions with a high specificity for the sensory modality of the information considered (e.g., visual vs. auditory), but also areas that are involved in more general cognitive processes, and especially linked to long-term memory storage, such as middle temporal lobes, inferotemporal gyri and the striatum^76^. Although more studies are needed to reach a deeper understanding of possible shared mechanisms, at present the anatomical overlap between the supra-modal structures involved in predictive coding and statistical learning seems to be rather limited. It is possible therefore that the perceptual, cognitive and behavioral phenomena associated with the two might arise from different types of computations, with statistical learning relying to a much greater degree on long-term memory traces.

Overall, a fundamental asymmetry seems to emerge between the mechanisms supporting selection vs. suppression history^18,77^. While the effects of selection history (which involve episodes comprehensive of stimulus selection, discrimination and response selection) can endure in time, and shape performance in future encounters with the same stimuli, in line with other forms of statistical learning, those associated with pure suppression history seem more labile, and readily adjustable according to changed circumstances, possibly following predictive computations aiding attentional and behavioral deployment. Indeed, these two aspects of cognition stem from very different needs in order to deal efficiently with the environment. While there are no obvious pitfalls in storing lasting traces of successful encounters with information that in the past has been attended to because relevant for the ongoing processing, a symmetrical and opposite treatment for salient distractors might be less convenient. In fact, from a practical point of view the possibility of changing systematically and permanently the sensitivity towards salient irrelevant events because they occur at a location previously associated with distraction seems to be more harmful than useful. In everyday life salient stimuli in fact carry environmental information of paramount importance, that makes it vital to not exclude a priori their processing. So, while past experience may help shape their impact within a moment-to-moment timescale, no traces are stored in the long term with respect to these adjustments, because there is an intrinsic value in being subject to distraction.

## Materials and methods

The study was carried out in accord with the WMA Declaration of Helsinki and with APA ethical standards, and it was approved by the Review Board for Research involving Human Participants of the University of Verona (protocol number: 2018-UNVRCLE-0272489). Participants were healthy volunteers recruited among the student population at the University of Verona, with normal or corrected to normal visual acuity and naïve to the purpose of the experiments. All of them signed an informed consent form before taking part in the study and received a fixed monetary compensation at the end of the last experimental session (€20 in Experiments 1 and 3, €15 in Experiment 2). Each subject took part in only one of the experiments in this study and had never participated before in similar or related experiments. All raw data were initially inspected in order to exclude trials in which, upon the presentation of the relevant stimuli, the participant either made and eyeblink or was not maintaining fixation. In some participants the number of trials falling into this category was so high that this filtering procedure led to missing data in the relevant experimental conditions. The data of these subjects had therefore to be excluded from the statistical analyses.

### Experiment 1

#### Participants

Twenty-one participants were initially recruited for the study, but three of them had to be excluded because of a large amount of missing data. The final sample, therefore, comprised 18 participants (6 males; mean age 22 years ± 3.2 SD).

#### Apparatus, stimuli and task

The experiment was programmed and run on OpenSesame 3.1.4^78^ using PsychoPy^79^ as a back-end and PyGaze^80^ as an interface for eye-tracking devices. The visual displays were presented on a 24-inch BenQ XL2430T LCD monitor, with a resolution of 1920 × 1080 pixels and a refresh rate of 144 Hz. Acoustic tones, such as error signals, were delivered through loudspeakers connected to the PC. The eye-movements of the right eye only were collected and recorded by the SR Research Eyelink 1000 Plus desktop-mounted system, with a 1000 Hz temporal and 0.01° spatial resolution. Participants were tested in a quiet and dimly lit room. Head movements were constrained with a chin-rest at a viewing distance of 57 cm from the display. At the start of each experimental session a 9-point calibration procedure ensured the correct reading of eye-position. The task employed was the same used in our previous work^38^ (see Fig. 1 for an illustration of the task).

All trials began with an eye drift correction trial in which participants had to fix a central white dot (1.25° in diameter) presented on a uniform dark gray background (luminance: 14.1 cd/m^2^). Following fixation, upon the onset of the stimulus display, participants were free to move their eyes elsewhere. Six grey circles (luminance: 68.6 cd/m^2^; 2.5° in diameter), with a pre-mask consisting of a grey asterisk located inside (luminance: 39 cd/m^2^; 0.4° in size), were simultaneously presented at 10° of eccentricity, equally spaced at the 1, 3, 5, 7, 9 and 11 o’clock positions of an imaginary circle. After a random variable interval between 500 and 800 ms, the fixation dot was removed and all the circles changed color, becoming green (CIE chromaticity coordinates: x = 0.154, *y* = 0.590; luminance: 68.2 cd/m^2^), except for the singleton stimulus acting as the target, which was the only one remaining grey. At the same time, all the asterisks inside the circles were removed, unveiling a left- or right-tilted small grey line (luminance: 39 cd/m^2^; 0.4° long and 0.06° wide, tilted by 30° from the vertical). This search display was available until response or for 1000 ms. The task demand was to provide a manual response reporting whether the target line was tilted to the left or to the right by pressing the “N” or “M” key on a QWERTY keyboard. Participants were encouraged to be as fast and accurate as possible. If the discrimination response was incorrect an error display appeared, accompanied by an 800 Hz tone. In a given proportion of trials (see below for details) an additional red circle (CIE chromaticity coordinates: x = 0.599, *y* = 0.372; luminance: 60.8 cd/m^2^) appeared abruptly in the display at one of the empty locations between the other circles. This stimulus stood out and was particularly salient both because of its unique color, and because of its sudden onset. Stimuli with these properties attract attention and eye-gaze in a reflexive way, and when irrelevant to the task, such as in this case, become salient distractors and give rise to remarkable costs in performance^38,50,54^.

#### Procedure

Experiment 1 was performed on two consecutive days (Fig. 2a). On the first day, after a brief practice block of 16 trials, participants performed the Baseline phase (144 trials) in which the distractor was present in 50% of the trials and appeared randomly and equally across six possible locations. The second phase, regarded as Training (900 trials) started immediately after the end of the Baseline block, in a seamless manner. During Training the distractor was present in 64% of the trials overall, and its distribution across locations was unbalanced by design as follows. Two locations, one for each hemifield and counterbalanced across participants, were occupied by the distractor with High Frequency (HF; overall 76% of the distractor present trials, 38% for each location); at the remaining four it appeared with Low Frequency (LF; 24% of the distractor present trials, 6% for each location). In line with our previous work^38^, for the purpose of statistical analyses only two out of the four total LF locations were considered as a counterpart to HF locations, those occupying the mirroring position on the opposite hemifield.

During the whole experimental session, which lasted about 90 min, participants could take a short break if needed after every ~ 50 trials. On the second day, participants performed in a Test phase (504 trials), the procedure of which was identical to the Baseline, with the salient distractor appearing in 50% of trials and no unbalances in its location probability. This session lasted approximately 45 min and a short break if needed could be taken after every ~ 50 trials.

While the probability of distractor location was biased by design during the Training phase as explained, throughout the whole Experiment the position of the target was equally likely across the six possible target locations. As can be clearly ascertained (Fig. 2a), the spatial locations that could be occupied by targets and distractors were independent of one another.

#### Data analysis

Statistical analyses were performed by using R 3.6.3^81^ and the additional package Rmisc^82^ on the first saccades detected from the onset of the search array considering only trials in which subjects responded correctly in the target discrimination task. Saccades were detected whenever an eye movement exceeded the velocity threshold of 35°/s with a minimum acceleration of 9.5°/s. Saccadic latency was defined as the interval between the onset of the search array and the beginning of the saccadic eye-movement. These were then considered in the analyses if their latency was comprised between 60 and 800 ms (this criterion led to the exclusion of 3.3% of trials). Saccades were labeled according to the landing position they reached within the search display. Specifically, we considered as target-directed saccades those that fell within the slice-shaped display area within 20° from the target (i.e., + 20 or − 20 angular degrees, vertex placed at the center of the display). On the other hand, we considered as distractor-directed saccades those in which the endpoint fell within a slice area of the display around the salient distractor. The width of this area was adjusted according to the distance between the distractor and the target in the given trial: within 10° from distractors that appeared at 30° from the target; 45° from distractors appearing at 90° from the target; 90° from distractors located 150° away from the target^83^. The dependent variables in all of the main statistical analyses performed were the percentages of target- and distractor-directed saccades in the experimental conditions of interest.

### Experiment 2

#### Participants

Twenty-four participants were initially recruited for the study, but four of them had to be excluded for missing data. The final sample, therefore, comprised 20 participants (10 males; mean age 22 years ± 3.2 SD).

#### Apparatus, stimuli and task

These were identical to those adopted in Experiment 1.

#### Procedure

The procedure was the same as Experiment 1 except for the fact that the three experimental phases took part on the same day, within a single experimental session (Fig. 2b). The whole Experiment comprised overall 1054 trials (Practice: 16; Baseline: 72; Training: 750; Test: 216) and lasted approximately 2 h.

#### Data analysis

The approach adopted for the preprocessing of raw data was the same as in Experiment 1. The filtering criteria applied to eye-movements on the basis of their latency led to discarding 2% of the trials.

### Experiment 3

#### Participants

Twenty participants were initially recruited for the study, but one of them had to be excluded because of missing data. The final sample, therefore, comprised 19 participants (8 males; mean age 25 years ± 4.5 SD).

#### Apparatus, stimuli and task

These were identical to those adopted in Experiments 1 and 2.

#### Procedure

The procedure was the same as in Experiment 1, and the only—yet crucial—difference consisted in the stimuli that were associated with frequency biases during the Training phase. In this experiment the salient distractor appeared in 50% of trials and when present could occupy equally often each of the six possible distractor locations. The target instead appeared more often at two specific locations in the display, one in each hemifield and counterbalanced across participants (Fig. 2c). The entity of the unbalance between high and low frequency locations was similar to the one applied to distractors in Experiments 1 and 2, therefore each high frequency location was occupied by the target on 38% of trials (overall the target probability in high frequency locations was 76%), while each low frequency location was associated with a 6% probability of hosting a target (leading to an overall probability of 24%). As in Experiment 1, on the first day participants performed a brief Practice block (16 trials), the Baseline (144 trials) and Training (768 trials) phases. On the following day they performed a Test session (504 trials) in which all the frequency unbalances that had been present during Training were removed.

#### Data analysis

The adoption of the same criteria for the preprocessing of raw data in Experiments 1 and 2 led to discarding 2% of the trials.

## Supplementary Information

Supplementary Information.

## Data Availability

The datasets generated and analysed during the current study are available from the Authors on motivated request.
