# Supplementary material for: Statistical learning of target selection and distractor suppression shape attentional priority according to different timeframes
Source: Sci Rep. 2021 Jul 2;11:13761. doi: 10.1038/s41598-021-93335-0 (PMC8253746; doi:10.1038/s41598-021-93335-0)
Supplement: Supplementary file 1 — Supplementary Information. [file 41598_2021_93335_MOESM1_ESM.docx]

**Statistical learning of target selection and distractor suppression shape attentional priority according to different timeframes.**

Valeria Di Caro, & Chiara Della Libera

Department of Neurosciences, Biomedicine and Movement Sciences, University of Verona, Verona, Italy

**SUPPLEMENTARY MATERIALS**

**Address for correspondence:**

Chiara Della Libera, PhD

Department of Neurosciences, Biomedicine and Movement Sciences

Section of Physiology and Psychology, University of Verona – Medical School,

Strada Le Grazie 8, 37134 Verona, Italy

Telephone: +39-045-8027219

Email: [chiara.dellalibera@univr.it](mailto:chiara.dellalibera@univr.it)

**SUPPLEMENT 1: Statistical analyses of distractor-directed saccades**

**EXPERIMENT 1**

**Baseline.** Distractor-directed saccades at baseline were compared across conditions in which distractors appeared at locations that would later be associated with high or low frequency. Unexpectedly, this analysis led to a statistically significant effect, although these specific locations were counterbalanced across subjects. The direction of this spurious effect was however in the opposite direction with respect to what would be expected following Training, HF 32.2% (±4.21), LF 26.4% (±4.18), *t*(17) = 2.291, *p* = 0.035, *d_z_* = 0.540 (Figure S1.1a).


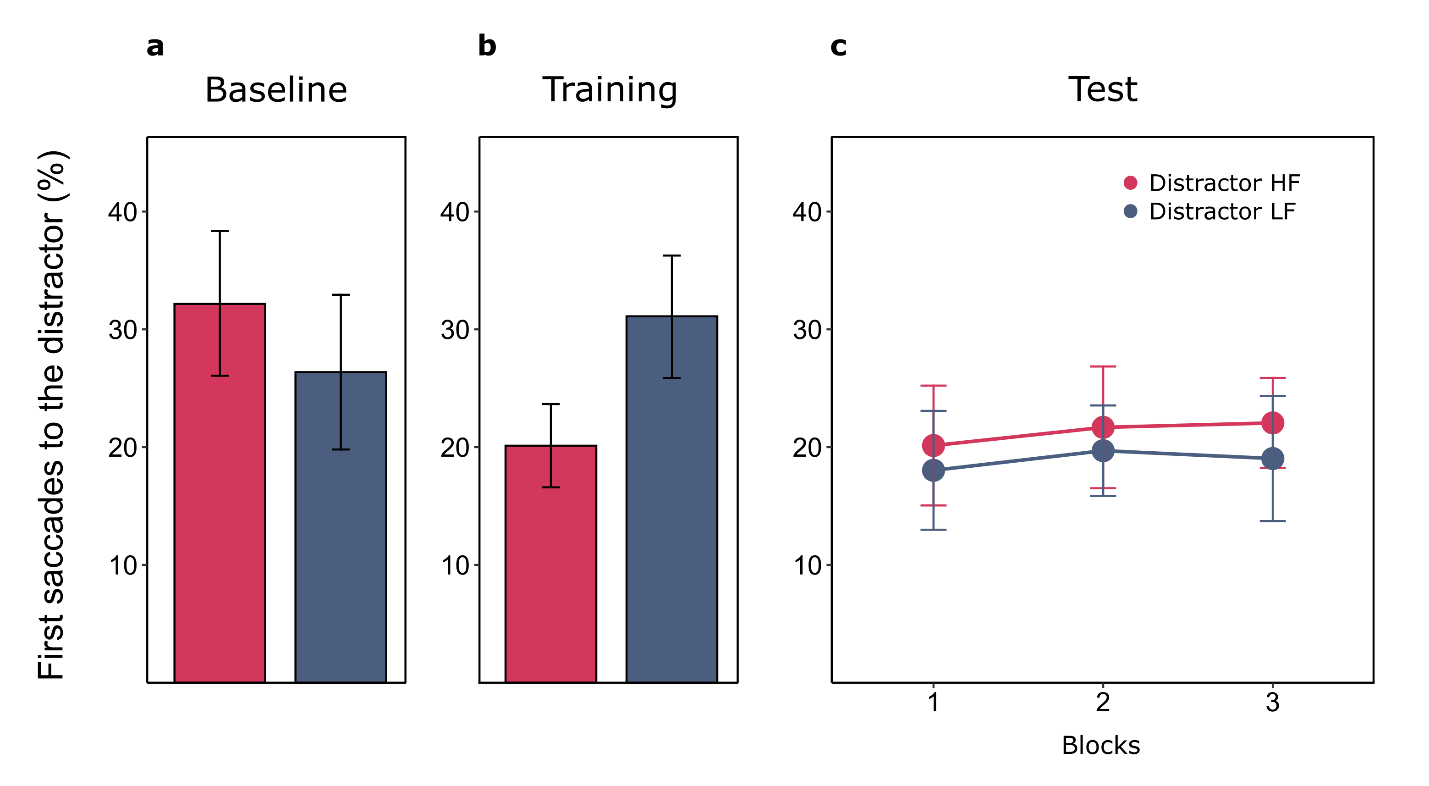


**Figure S1.1.** Oculomotor capture in Experiment 1: Mean percentage of distractor-directed saccades as a function of Distractor location. **a)** Performance at Baseline. **b)** Performance at Training. **c)** Performance at Test. Here and in all the other Supplementary Figures, error bars depict the within-subject confidence intervals.

**Training phase.** The percentage of first saccades directed towards the distractor was significantly lower when it appeared at a HF location, HF 20.1% (±2.99), LF 30.9% (±4.81), *t*(17) = 4.735, *p* < 0.001, *d_z_* = 1.116, suggesting, in line with our previous research, that suppression history at these locations rendered them less likely to attract attention and eye-gaze even when a highly salient event appeared therein. (Figure S1.1b).

**Test phase.** The Test phase was divided in three consecutive blocks and the ANOVA carried out on distractor-directed saccades considered Distractor location (HF vs. LF) and Block (1 to 3) as main factors. No significant effects emerged from this analysis (Distractor location: *F*(1,17) = 1.469, *p* = 0.242, ƞ_p_^2^ = 0.080; Block: *F*(2,34) = 0.439, *p* = 0.648, ƞ_p_^2^ = 0.025; Distractor location x Block: *F*(2,34) = 0.076, *p* = 0.927, ƞ_p_^2^ = 0.004) (Figure S1.1c).

**Training vs. Test.** Changes in performance from Training to Test were examined by means of an ANOVA with Phase (Training vs. Test) and Distractor location (HF vs. LF) as main factors. The main effect of Phase was significant, Training 25.5% (±3.84), Test 20.1% (±2.73), *F*(1,17) = 5.244, *p* = 0.035, ƞ_p_^2^ = 0.236, indicating a remarkable reduction in the oculomotor capture associated with the Distractor on the second day. The main effect of Distractor location was also significant, HF 20.7% (±2.83), LF 24.9% (±3.51), *F*(1,17) = 9.503, *p* = 0.007, ƞ_p_^2^ = 0.359, however it also interacted significantly with Phase, *F*(1,17) = 16.431, *p* < 0.001, ƞ_p_^2^ = 0.491. Indeed, as had already emerged in the analyses within each phase, the effect of suppression history was eliminated at Test (Distractor location effect at Training 5.41% (±1.14) vs. at Test -1.19% (0.98), *t*(17) = 4.054, *p* < 0.001, *d_z_* = 0.955).

The changes across phases were further examined comparing performance at Training with the first Test Block, showing that while oculomotor capture associated with distractors in HF locations was low and comparable in Training and Test, 20.1% (±2.99) and 20.1% (±2.94), *t*(17) = 0.009, *p* = 0.993, *d_z_* = 0.002, it was dramatically reduced – at Test – for those appearing at LF locations, 30.9% (±4.81) and 18.0% (±3.09), *t*(17) = 3.677, *p* = 0.002, *d_z_* = 0.867.

**EXPERIMENT 2**

**Baseline.** No significant differences were found with respect to trials in which distractors appeared at locations that would later become associated with high or low frequency, *t*(19) = 0.695, *p* = 0.496, *d_z_* = 0.155) (Figure S1.2a).


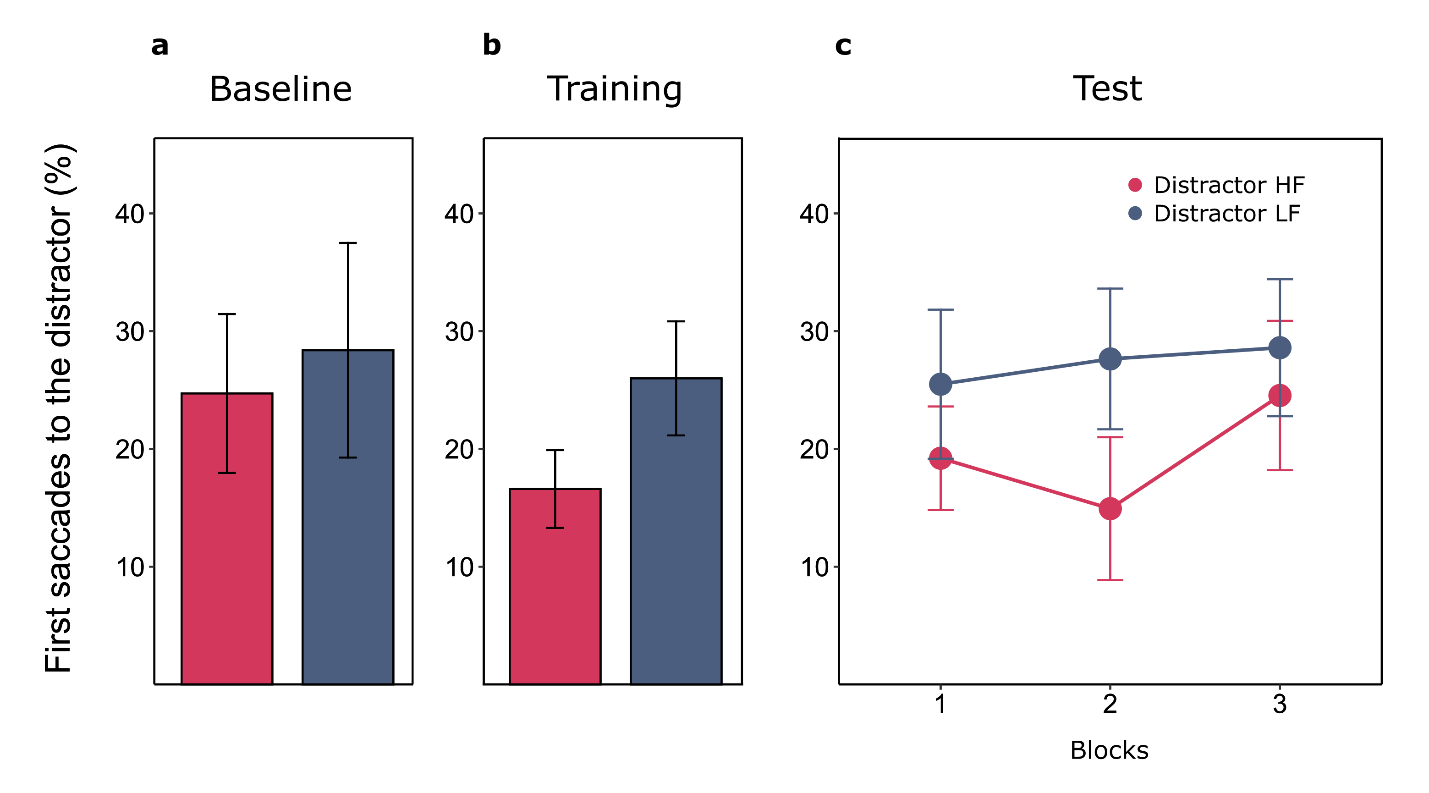
**Figure S1.2.** Oculomotor capture in Experiment 2: Mean percentage of distractor-directed saccades as a function of Distractor location. **a)** Performance at Baseline. **b)** Performance at Training. **c)** Performance at Test.

**Training phase.** Once again, distractors appearing at HF locations received a significantly lower amount of first saccades relatively to those at LF locations, HF 16.6% (±1.95), LF 26.2% (±2.85), *t*(19) = 5.843, *p* < 0.001, *d_z_* = 1.306 (Figure S1.2b).

**Test phase.** The ANOVA with Distractor location and Block as factors revealed a significant main effect of Distractor location, HF 19.6% (±2.26), LF 27.2% (±3.15), *F*(1,19) = 14.722, *p* = 0.001, ƞ_p_^2^ = 0.437. The main effect of Block, *F*(2,38) = 1.918, *p* = 0.161, ƞ_p_^2^ = 0.092, and the Block by Distractor location interaction were instead not significant, *F*(2,38) = 1.856, *p* = 0.170, ƞ_p_^2^ = 0.089) (Figure S1.2c).

**Training vs. Test.** The ANOVA conducted across phases revealed a significant main effect of Distractor location, HF 18.1% (±1.90), LF 26.7% (±2.65), *F*(1,19) = 39.036, *p* < 0.001, ƞ_p_^2^ = 0.673, while both the main effect of Phase, *F*(1,19) = 0.929, *p* = 0.347, ƞ_p_^2^ = 0.047, and its interaction with Distractor location were not significant, *F*(1,19) = 0.624, *p* = 0.439, ƞ_p_^2^ = 0.032).

**EXPERIMENT 3**

**Baseline.** No significant effects were found in distractor directed saccades considering trials in which the target appeared at the locations that would become later associated with frequency manipulations, *t*(18) = 0.048, *p* = 0.962, *d_z_* = 0.011 (Figure S1.3a).


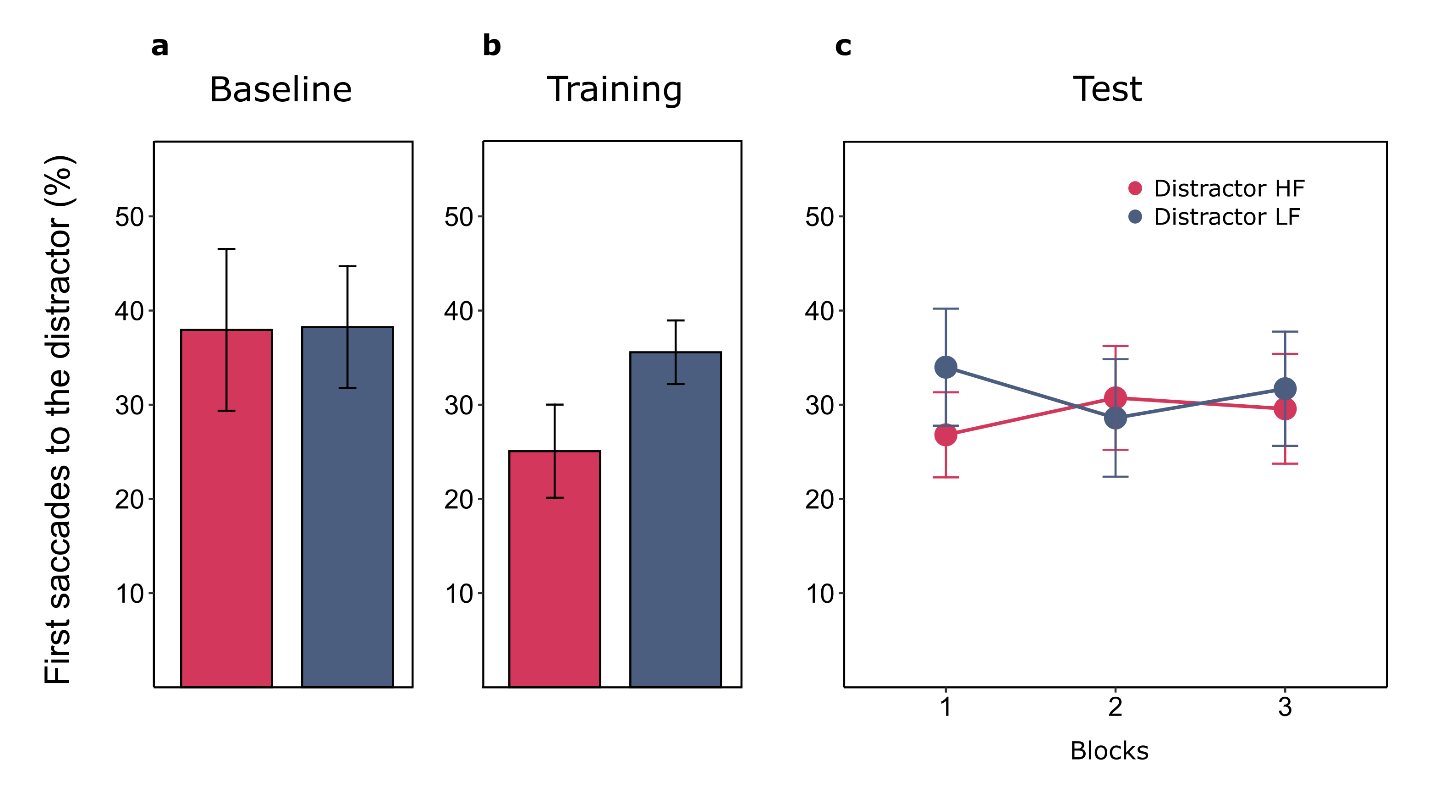


**Figure S1.3.** Oculomotor capture in Experiment 3: Mean percentage of distractor-directed saccades as a function of Target location. **a)** Performance at Baseline. **b)** Performance at Training. **c)** Performance at Test.

**Training phase.** A direct contrast was performed to compare distractor-directed saccades in trials with targets appearing at HF vs. LF locations. The result of this test reinforced the notion that, if a target appeared at a HF location, the salient distractor elicited a lower degree of oculomotor capture, HF 25.0% (±3.93), LF 36.1% (±3.79), *t*(18) = 4.747, *p* < 0.001, *d_z_* = 1.089 (Figure S1.3b).

**Test phase.** None of the effects of the ANOVA with Target location and Block as main factors was significant (Target location: *F*(1,18) = 1.026, *p* = 0.324, ƞ_p_^2^ = 0.054; Block: *F*(2,36) = 0.079, *p* = 0.924, ƞ_p_^2^= 0.004; Target location x Block: *F*(2,36) = 1.348, *p* = 0.273, ƞ_p_^2^ = 0.070) (Figure S1.3c).

**Training vs. Test.** The ANOVA performed comprised Phase, Target location and Distractor presence as within-subjects effects. The main effects of Target location was significant, in line with the lower effect of oculomotor capture when Targets appear at HF locations, which was also found in the original ANOVAs, HF 27.0% (±4.07), LF 33.7% (±3.39), *F*(1,18) = 14.518, *p* = 0.001, ƞ_p_^2^ = 0.446. The main effect of Phase was non-significant, *F*(1,18) = 0.036, *p* = 0.852, ƞ_p_^2^ = 0.002; while the interaction between the two was significant, *F*(1,18) = 7.961, *p* = 0.011, ƞ_p_^2^ = 0.307, suggesting that the overall impact of target location was significantly lower during the Test phase, Target location effect at Training 11.12 ms (±2.34), at Test 2.39 ms (±2.36).

**SUPPLEMENT 2: Statistical analyses of Reaction Times in manual task responses**

All analyses were conducted on the Reaction Times (RTs) of correct responses to the task. Task accuracy was very high in every Experiment (around 98%) and was not analyzed as a dependent variable.

**EXPERIMENT 1**

**Baseline.** Task responses were significantly slower in trials in which the salient distractor was present, absent 781 ms (±16.1), present 832 ms (±16.9), *t*(17) = 10.331, *p* < 0.001, *d_z_* = 2.435. No significant differences were found associated with distractor location, *t*(17) = 0.164, *p* = 0.871, *d_z_* = 0.039 (Figure S2.1a).


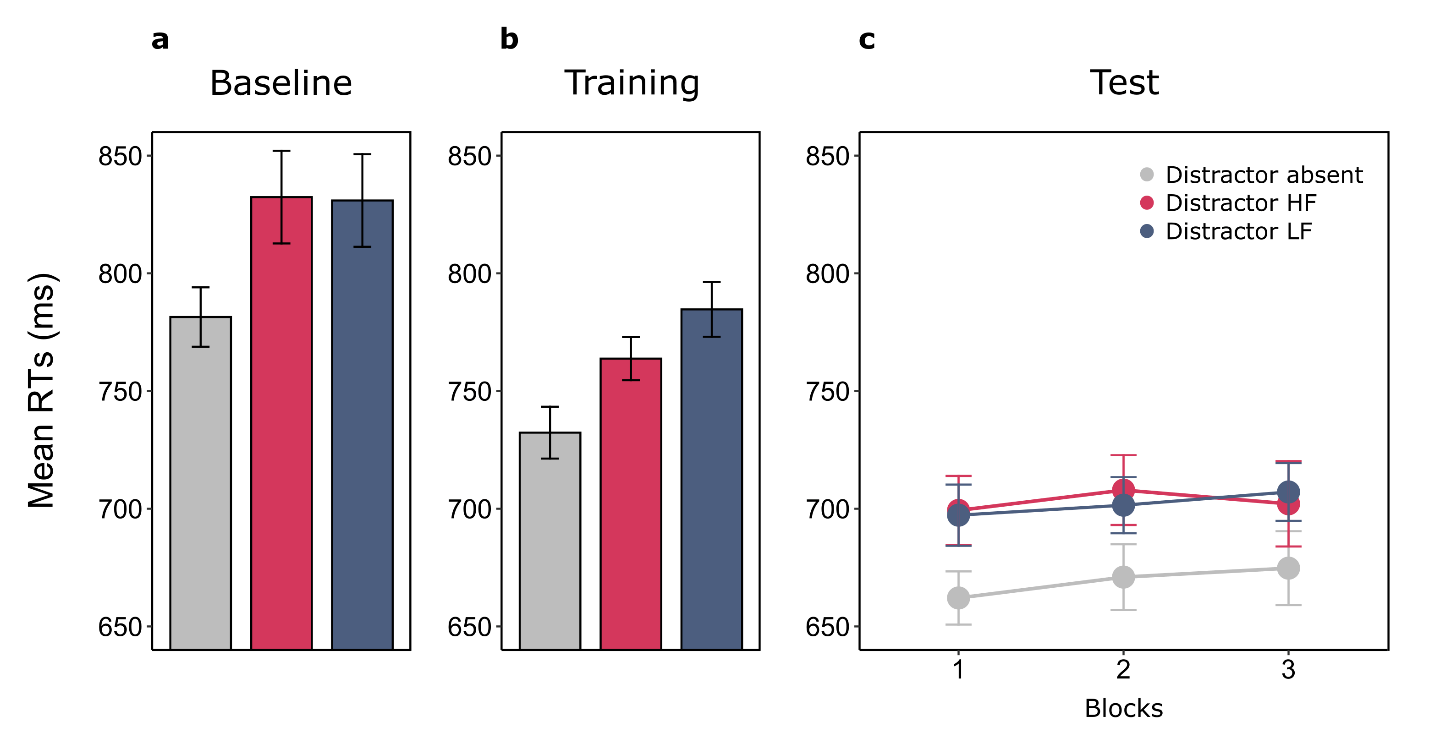


**Figure S2.1.** Correct responses in the manual discrimination task of Experiment 1: Mean RTs as a function of Distractor location. **a)** Performance at Baseline. **b)** Performance at Training. **c)** Performance at Test.

**Training phase.** The mean RTs of task responses were submitted to a one-way ANOVA with Distractor location as main factor (absent, present in HF location, present in LF location). The effect of Distractor location was significant, absent 733 ms (±17.5), HF 764 ms (±16.9), LF 784 ms (±18.1), F*(*2,34) = 41.213, *p* < 0.001, ƞ^2^ = 0.708, and post-hoc t-tests confirmed that while a salient distractor slowed manual responses in general (absent vs. HF: *F*(17) = 6.888, *p* < 0.001, *d_z_* = 1.624; absent vs. LF: *t*(17) = 8.119, *p* < 0.001, *d_z_* = 1.914, the impact of those appearing where distraction was more likely was significantly lower (HF vs. LF: *t*(17) = 3.299, *p* = 0.004, *d_z_* = 0.777) (Figure S2.1b).

**Test phase.** The Test phase was divided in three consecutive blocks, and an ANOVA was conducted with Distractor location (absent, HF, LF) and Block (1 to 3) as within-subjects factors. The effect of Distractor location was significant, absent 669 ms (±12.9), HF 703 ms (±13.8), LF 702 ms (±14.0), *F*(2,34) = 49.448, *p* < 0.001, ƞ_p_^2^ = 0.744, and post-hoc t-tests suggested, surprisingly, that while distractors always impaired performance (absent vs. HF: *t*(17) = 8.093, *p* < 0.001, *d_z_* = 1.907; absent vs. LF: *t*(17) = 8.158, *p* < 0.001, *d_z_* = 1.923), the difference between the two crucial conditions was no longer significant (HF vs. LF: *t*(17) = 0.339, *p* = 0.739, *d_z_* = 0.080) (Figure S2.1c). Neither Block, *F*(2,34) = 0.929, *p* = 0.405, ƞ_p_^2^ = 0.052, nor its interaction with Distractor location, *F*(4,68) = 0.546, *p* =0.703, ƞ_p_^2^ = 0.031, were significant.

**Training vs. Test.** To compare directly Training and Test phases we conducted an ANOVA with Phase (Training vs. Test) and Distractor location (absent, HF, LF) as factors. The overall effect of Phase was significant, Training 760 ms (±17.2), Test 691 ms (±13.4), *F*(1,17) = 103.996, *p* < 0.001, ƞ_p_^2^ = 0.859, indexing an overall reduction of RTs as a function of practice. In line with the separate analyses for each phase, the effect of Distractor location was also significant, absent 701 ms (±14.9), HF 733 ms (±14.8), LF 743 ms (±15.8), *F*(2,34) = 61.749, *p* < 0.001, ƞ_p_^2^ = 0.784, and interacted significantly with Phase, *F*(2,34) = 8.364, *p* = 0.001, ƞ_p_^2^ = 0.330). Indeed, the effect of Distractor presence changed dramatically across phases, so that the overall impact of salient distractors was less detrimental on the second day, Distractor presence effect during Training 41.3 ms (±4.59), during Test 33.3 ms (±3.74), *t*(17) = 2.184, *p* = 0.043, *d_z_* = 0.515. Moreover, the change in the effect associated with distractor location was also reduced at Test (HF vs. LF during Training -19.99 ms (±6.06), during Test 1.14 ms (±3.35), *t*(17) = 3.127, *p* = 0.006, *d_z_* = 0.737).

Comparing performance at Training with Test block 1, separately for trials with distractors in HF and LF locations we found significant effects in both cases, indicating that even on the very first part of the Test session performance associated with distractors in either location was more efficient with respect to the previous day (HF during Training 764 ms (±16.9), during Test block 1 699 ms (±15.9): *t*(17) = 7.153, *p* < 0.001, *d_z_* = 1.686; LF during Training 784 ms (±18.1), during Test block 1 697 ms (±14.1): *t*(17) = 9.211, *p* < 0.001, *d_z_* = 2.171).

**EXPERIMENT 2**

**Baseline.** Distractors delayed responses significantly at baseline, absent 761 ms (±15.6), present 813 ms (±17.4), *t*(19) = 7.441, *p* < 0.001, *d_z_* = 1.664. No significant differences were found in trials with distractors at locations later associated with high or low frequency (HF vs. LF: *t*(19) = 1.121, *p* = 0.276, *d_z_* = 0.251) (Figure S2.2a).

**Training phase.** The one-way ANOVA revealed a significant main effect of Distractor location, absent 704 ms (±16.0), HF 734 ms (±16.5), LF 757 ms (±18.7), *F*(2,38) = 66.453, *p* < 0.001, ƞ^2^ = 0.778, and post-hoc t-tests replicated exactly the results of the Training phase in Experiment 1, once again demonstrating that task performance, while being always impaired by the presence of a distractor (absent vs. HF: *t*(19) = 9.175, *p* < 0.001, *d_z_* = 2.052; absent vs. LF: *t*(19) = 9.247, *p* < 0.001, *d_z_* = 2.068), was significantly better when this appeared at HF locations (HF vs. LF: *t*(19) = 5.050, *p* < 0.001, *d_z_* = 1.129) (Figure S2.2b).

**Test phase.** The ANOVA on task responses during the Test phase highlighted a significant main effect of Distractor location, absent 713 ms (±18.7), HF 748 ms (±18.0), LF 757 ms (±19.0), *F*(2,38) = 33.230, *p* < 0.001, ƞ_p_^2^ = 0.636. The main effect of Block, *F*(2,38) = 0.373, *p* = 0.691, ƞ_p_^2^ = 0.019, and the interaction between Block and Distractor location were instead non-significant, *F*(4,76) = 1.018, *p* = 0.404, ƞ_p_^2^ = 0.051 (Figure S2.2c). So, distractors always had a significant impact on performance (absent vs. HF: *t*(19) = 5.839, *p* < 0.001, *d_z_* = 1.306; absent vs. LF: *t*(19) = 8.685, *p* < 0.001, *d_z_*=1.942). Here however, the advantage associated with distractors at HF locations, compared to LF, was no longer statistically significant (HF vs. LF: *t*(19) = 1.470, *p* = 0.158, *d_z_* =0.329).


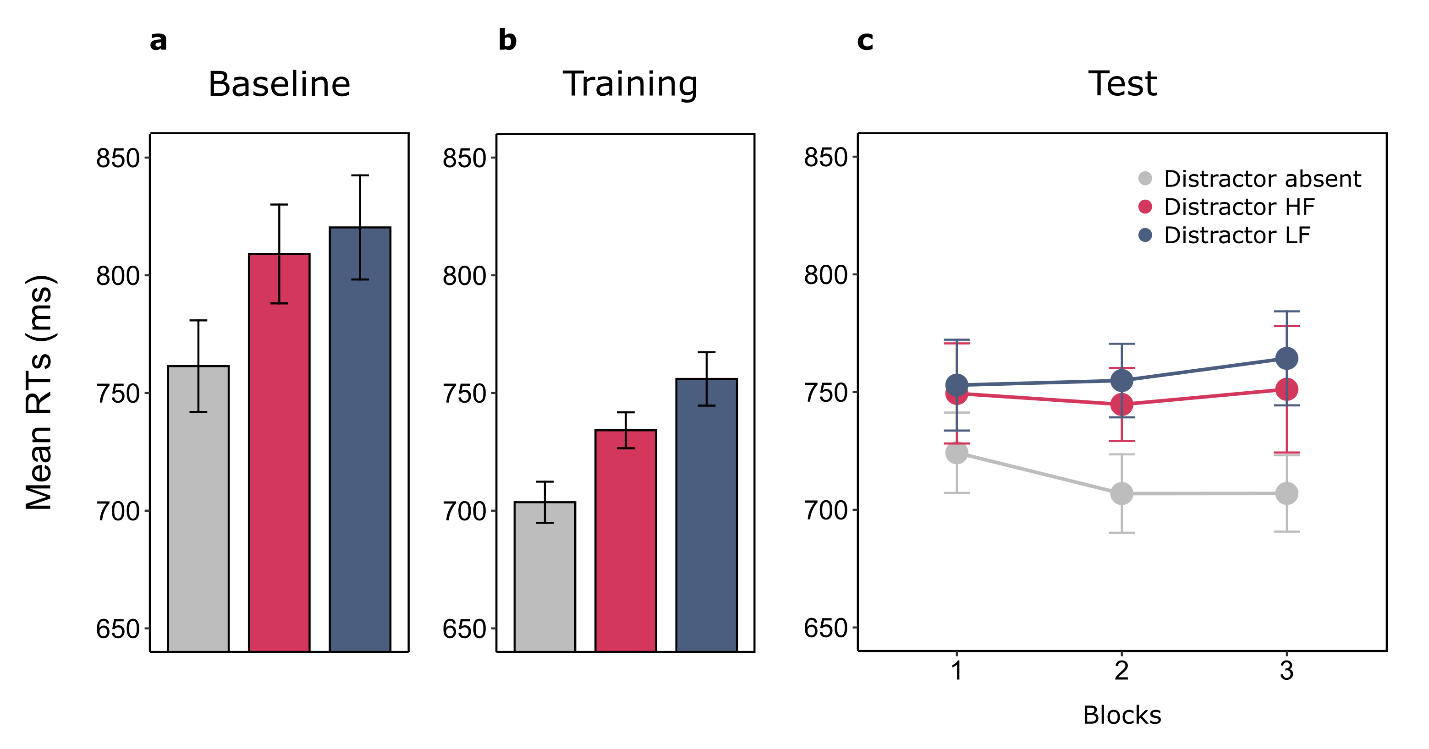


**Figure S2.2.** Correct responses in the manual discrimination task of Experiment 2: Mean RTs as a function of Distractor location. **a)** Performance at Baseline. **b)** Performance at Training. **c)** Performance at Test.

**Training vs. Test.** The ANOVA with Phase and Distractor location as factors resulted in a significant effect of Distractor location, absent 708 ms (±17.0), HF 741 ms (±16.6), LF 757 ms (±18.2), *F*(2,38) = 78.685, *p* < 0.001, ƞ_p_^2^ = 0.805, and non-significant effects of Phase, *F*(1,19) = 0.915, *p* = 0.351, ƞ_p_^2^ =0.046, and Distractor location by Phase, *F*(2,38) = 1.960, *p* = 0.155, ƞ_p_^2^ = 0.094. Interestingly, the differential impact of distractor at locations previously associated with a different suppression history did not reach statistical significance (HF vs. LF effect across Phases: *t*(19) = 1.737, *p* = 0.099, *d_z_* = 0.388).

Direct tests between performance at Training and during the very first Block of the Test phase indicated that the difference between performance associated with HF and LF distractors, respectively, was not significantly different across phases (HF in Training vs. Test Block 1: *t*(19) = 1.242, *p* = 0.229, *d_z_* = 0.278; LF in Training vs. Test Block 1: *t*(19) = 0.333, *p* = 0.743, *d_z_* = 0.074).

**EXPERIMENT 3**

**Baseline.** The impact of distractors was significant, absent 777 ms (±27.7), present 837 ms (±25.6), *t*(18) = 5.961, *p* < 0.001, *d_z_* = 1.367, and no significant effects were found considering trials with targets appearing at locations later associated with frequency manipulations (HF vs. LF: *t*(18) = 0.599, *p* = 0.557, *d_z_* = 0.137) (Figure S2.3a).

**Training phase.** The ANOVA conducted considered Target location (HF vs. LF) and Distractor presence (present vs. absent) as within-subjects factors. The significant effect of Target location, HF 732 ms (±19.8), LF 769 ms (±20.2), *F*(1,18) = 69.658, *p* < 0.001, ƞ_p_^2^ = 0.795, indicated that targets at HF locations received significantly faster responses. The main effect of Distractor presence was also significant, absent 727 ms (±20.2), present 774 ms (±19.7), *F*(1,18) = 153.246, *p* < 0.001, ƞ_p_^2^ = 0.895, underlining once more the detrimental impact of distractors. The interaction between Target location and Distractor presence was also significant, *F*(1,18) = 5.097, *p* = 0.037, ƞ_p_^2^ = 0.221), indicating that while the effect of selection history was always significant, the advantage in performance associated with targets in HF locations was greater in the presence of a salient distractor, Target location effect in distractor absent 29.5 ms (±5.45) vs. present 43.8 ms (±5.37): *t*(18) = 2.258, *p* = 0.037, *d_z_* = 0.518 (Figure S2.3b).

**Test phase.** The ANOVA conducted on eye-movements at Test considered Target location (HF vs. LF), Distractor (absent vs. present) and Block (1 to 3) as within-subjects effects. Crucially, the main effect of Target location was significant, highlighting that selection history effects were maintained for at least one day after the learning session, HF 714 ms (±16.4), LF 723 ms (±17.8), *F*(1,18) = 5.317, *p* = 0.033, ƞ_p_^2^ = 0.228. The main effect of Distractor presence was also significant, absent 699 ms (±17.1), present 738 ms (±17.2), *F*(1,18) = 80.137, p < 0.001, ƞ_p_^2^ = 0.817. None of the other effects or interactions were significant (Block: *F*(2,36) = 3.102, *p* = 0.057, ƞ_p_^2^ = 0.147; Target location x Block: *F*(2,36) = 1.787, *p* = 0.182, ƞ_p_^2^ = 0.090; Target location x Distractor presence: *F*(1, 18) = 2.812, *p* = 0.111, ƞ_p_^2^ = 0.135; Block x Distractor presence: *F*(2,36) = 0.174, *p* = 0.841, ƞ_p_^2^ = 0.010; Target location x Block x Distractor presence: *F*(2,36) = 0.917, *p* = 0.409, ƞ_p_^2^ = 0.048) (Figure S2.3c).


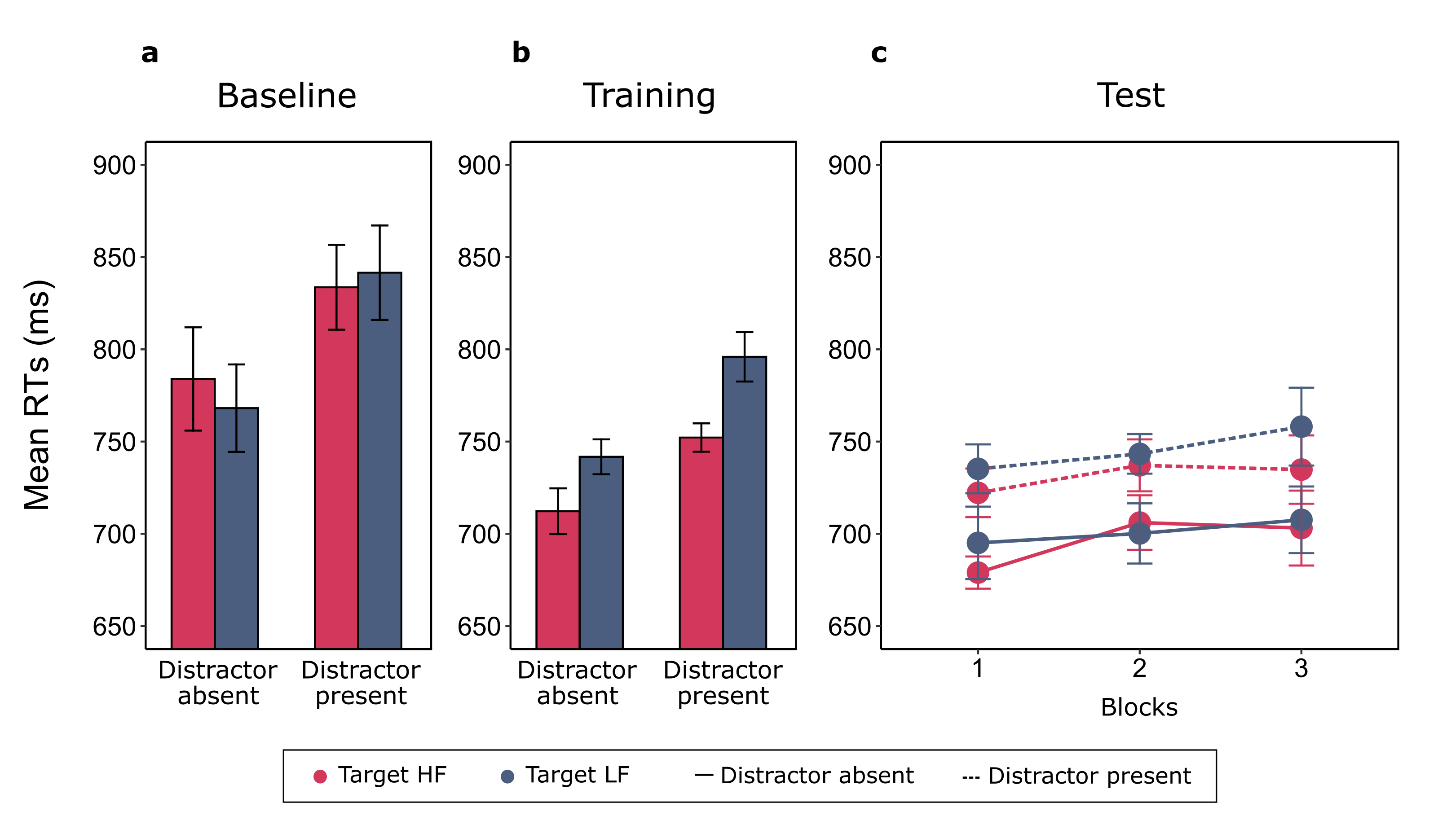


**Figure S2.3.** Correct responses in the manual discrimination task of Experiment 2: Mean RTs as a function of Target location and Distractor presence. **a)** Performance at Baseline. **b)** Performance at Training. **c)** Performance at Test.

**Training vs. Test.** The ANOVA comprised Phase, Target location and Distractor presence as within-subjects effects. The main effects of Phase was significant, revealing a robust decrease in RTs across sessions, Training 752 ms (±19.9), Test 718 ms (±17.0), *F*(1,18) = 39.137 *p* < 0.001, ƞ_p_^2^ = 0.685. The main effect of Target location was also significant, HF 723 ms (±17.9), LF 746 ms (±18.8), *F*(1,18) = 79.362, *p* < 0.001, ƞ_p_^2^ = 0.815, and so was its interaction with Phase, *F*(1,18) = 15.894, *p* < 0.001, ƞ_p_^2^ = 0.469, indicating that although the effect of selection history was still in place at Test, it became weaker, Target location effect at Training 36.6 ms (±4.39), at Test 10.1 ms (±4.08). The effect of Distractor presence was significant, absent 713 ms (±18.6), present 756 ms (±18.2), *F*(1,18)=136.621, *p* < 0.001, ƞ_p_^2^ = 0.884, and so was its interaction with Phase *F*(1,18) = 4.555, *p* = 0.047, ƞ_p_^2^ = 0.202, suggesting that the overall impact of distractors was significantly lower at Test, Distractor presence effect at Training 47.1 ms (±3.80), at Test 39.5 ms (±4.39).

The interaction between Target location and Distractor presence was also significant, *F*(1,18) = 11.786, *p* = 0.003, ƞ_p_^2^ = 0.396, indexing – in line with the separate analysis on Training – that in RTs to the main task the impact of selection history tended to be more visible in the presence of a salient distractor, Target location effect in distractor absent trials 7.95 ms (±2.64), in distractor present trials 16.51 ms (±3.99). Finally, the interaction comprising all of the three factors was non-significant, *F*(1,18) = 0.408, *p* = 0.531, ƞ_p_^2^ = 0.022.
